# Single-atom catalysts anchored on nanostructured frameworks for high-performance lithium–sulfur batteries: a review of catalytic mechanisms

**DOI:** 10.1039/d6ra05609a

**Published:** 2026-08-17

**Authors:** Xinbo Chang, Tao Li

**Affiliations:** a College of Materials Science and Engineering, Qingdao University of Science & Technology 266042 China; b College of Physical Education, Qingdao University of Science & Technology 266061 China litaoqdkjdx@163.com

## Abstract

Li–S batteries offer a theoretical specific capacity of 1675 mAh g^−1^ and specific energy near 2600 Wh kg^−1^, but practical cells remain limited by incomplete sulfur utilization, soluble lithium polysulfide (LiPS) shuttling, sluggish liquid–solid conversion, insulating Li_2_S/Li_2_S_2_, and lithium-metal instability. Single-atom catalysts (SACs) anchored on nanostructured frameworks combine high atom utilization and tunable coordination with conductive, porous architectures that regulate transport and confinement. This review examines SAC-based Li–S batteries by connecting site structure, catalytic mechanism, and cell-level performance. Unlike reviews organized mainly by metal identity or support class, it uses a mechanism-centered framework linking coordination and topology to adsorption, bidirectional conversion, Li_2_S growth, and practical metrics. It also distinguishes isolated-site SAC evidence from cluster and dual-atom comparators, evaluates descriptor and operando-validation limits, and identifies ultrathin bifunctional catalytic separators/interlayers as a near-term deployment route. Reported capacities, rates, sulfur loadings, areal capacities, electrolyte/sulfur ratios, cycle life, activation barriers, adsorption energies, Tafel slopes, and nucleation/decomposition metrics are compared in four comparative tables. The analysis shows that high apparent performance often depends on low sulfur loading, excess electrolyte, or coin-cell conditions, and that descriptors from idealized models must be interpreted with respect to product coverage, electrolyte chemistry, and site stability. SACs are most valuable when strong-but-not-immobilizing LiPS binding, fast electron/ion transport, controlled Li_2_S deposition, and reversible Li_2_S oxidation are achieved simultaneously. Future work should pair standardized lean-electrolyte, high-loading, limited–lithium, and pouch-cell testing with state-resolved validation of catalytic pathways and active-site stability.

## Abstract 2

This review examines SAC-based Li–S batteries by connecting site structure, catalytic mechanism, and cell-level performance.

## Graphical abstract

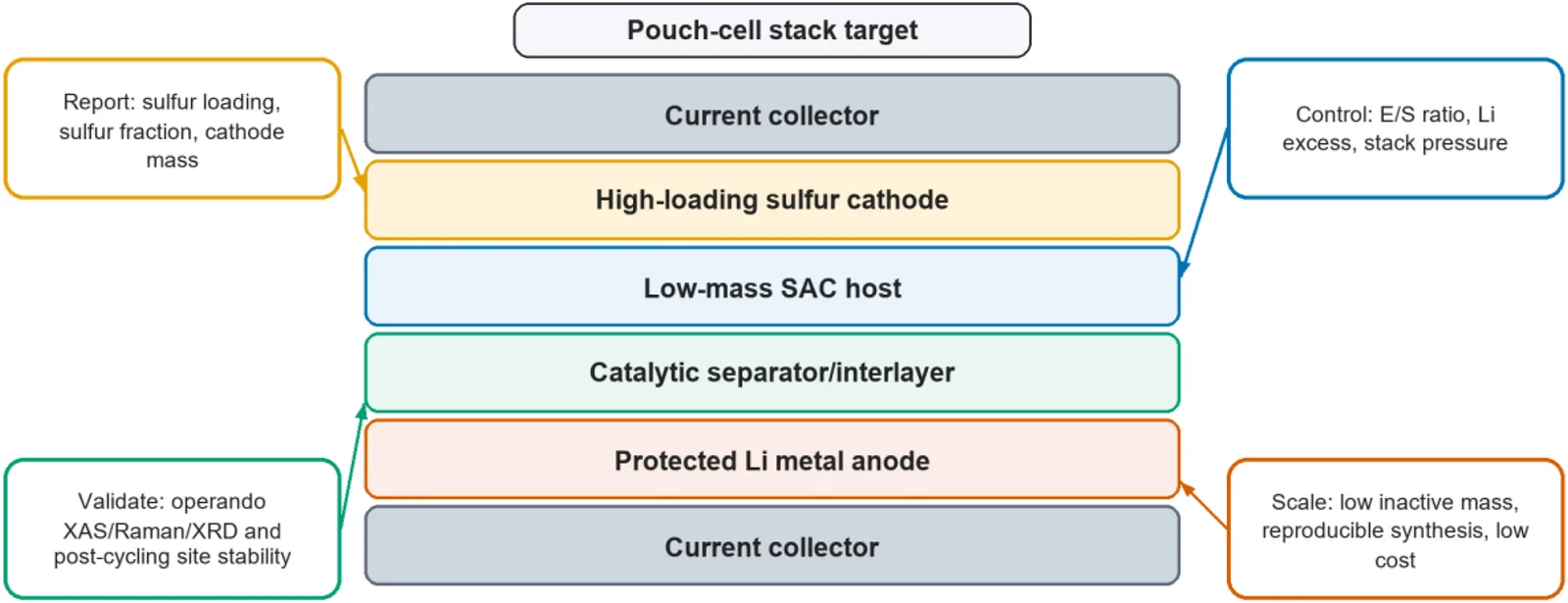

## 1. Introduction

Rechargeable Li–S batteries remain attractive because sulfur is abundant, inexpensive, and capable of multi-electron storage. The full conversion between S_8_ and Li_2_S corresponds to 16 electrons per sulfur molecule and gives a theoretical specific capacity of 1675 mAh g^−1^, which is several times higher than that of common intercalation cathodes.^1–3^ The chemistry is also relevant to engineering applications because the gravimetric energy target can, in principle, exceed conventional lithium-ion batteries if sulfur loading is high, electrolyte inventory is low, inactive host mass is controlled, and lithium excess is limited.^4–7^ These conditions are difficult to satisfy at the same time. The same conversion chemistry that gives Li–S cells their capacity also produces soluble Li_2_S_*x*_ intermediates, large volume change, electronically insulating solid products, and interfacial side reactions at lithium metal.^8–13^

The central cathode problem is not only the dissolution of LiPSs. It is the coupled failure of adsorption, diffusion, electron transfer, nucleation, and oxidation over a moving solid–liquid interface. During discharge, S_8_ first forms high-order soluble LiPSs, then lower-order LiPSs, and finally Li_2_S_2_/Li_2_S. The lower plateau contributes most of the theoretical capacity but is more kinetically demanding because soluble species must be converted into electronically insulating solids.^14–18^ During charge, Li_2_S and Li_2_S_2_ must be oxidized back to soluble intermediates and ultimately sulfur. A cathode host that only traps polysulfides can slow shuttle but can also immobilize intermediates away from conductive pathways. A conductive host that only provides physical confinement can leave the liquid–solid transformation slow. This is why catalytic hosts, separator coatings, and interlayers have been studied as reaction mediators rather than passive containers.^19–24^

Early nanostructured carbon hosts demonstrated that ordered mesoporosity, carbon conductivity, and nanoscale sulfur confinement can improve sulfur utilization and cycling stability.^25–28^ Polar oxides, sulfides, nitrides, carbides, MXenes, and phosphorene then added chemical adsorption and catalytic functions.^29–36^ These materials clarified several design rules. First, adsorption must be balanced with surface diffusion. Tao and co-workers^35^ showed that nonconductive oxides with very strong polysulfide adsorption can retard transfer to conductive carbon, whereas moderate adsorption and faster surface transport help form more uniform Li_2_S. Second, Li_2_S decomposition during charge is a catalytic step in its own right. Zhou and co-workers^36^ showed that metal sulfides such as TiS_2_ and VS_2_ can lower the Li_2_S decomposition barrier compared with carbon, explaining why hosts previously described as “anchors” can also serve as oxidation catalysts. Third, reaction kinetics under lean-electrolyte or high-loading conditions can differ from dilute, flooded coin cells, because solid product coverage changes the accessible catalyst surface.^37–40^

SACs add a further level of tunability. A single metal atom coordinated by N, O, S, P, or mixed heteroatoms can act as an electronically defined catalytic center. In principle, each metal atom is accessible, the coordination shell can be adjusted, and the electronic interaction with sulfur species can be described through adsorption energy, d-band position, charge transfer, spin state, or d–p orbital hybridization.^41–46^ In Li–S cells, the most common SAC motifs are M–N–C, M–N_*x*_–C, M–N/S–C, M–O/N–C, M–S–POP, and MOF-derived M–N–C frameworks. The isolated metal center binds LiPSs through metal–sulfur or metal–lithium interactions, while the framework provides conductivity, porosity, mechanical integrity, and electrolyte access.^47–53^

The design issue is therefore not whether SACs are active, but under what structural and electrochemical conditions their activity remains useful. A Co–N–C site in nitrogen-doped graphene can facilitate both Li_2_S formation and decomposition while enabling a sulfur composite with 90 wt% sulfur to deliver 1210 mAh g^−1^ and 5.1 mAh cm^−2^ at 6.0 mg cm^−2^ sulfur loading.^54^ A V single atom on nitrogen-doped graphene gives a calculated Li_2_S decomposition barrier of 1.10 eV, lower than graphene and several other single-metal sites, and supports improved rate capability under 5 mg cm^−2^ sulfur loading.^55^ More recent examples use asymmetric coordination, d–p orbital hybridization, high-loading separator coatings, and out-of-plane single-atom sites in porous organic polymers to accelerate conversion and suppress shuttle.^56–64^ These results are important, but they are not directly comparable unless sulfur loading, electrolyte/sulfur (*E*/*S*) ratio, catalyst loading, current density, voltage window, cycle count, and areal capacity are reported together.

Research published in 2025–2026 shows that the field is moving from single-metal screening toward controlled long–range interactions, explicit metal–support coupling, stability-aware high-throughput design, asymmetric Co–N_3_S_1_ coordination, single-atom/nanocluster relay catalysis, curvature-dependent dual-electrode regulation, and direct analysis of catalyst migration.^65–71^ These studies sharpen three questions that organize this review: whether isolated sites remain isolated during cycling, whether computed descriptors remain transferable under realistic solvation and product coverage, and whether catalytic gains justify the added inactive mass at the cell level.

**Fig. 1:**
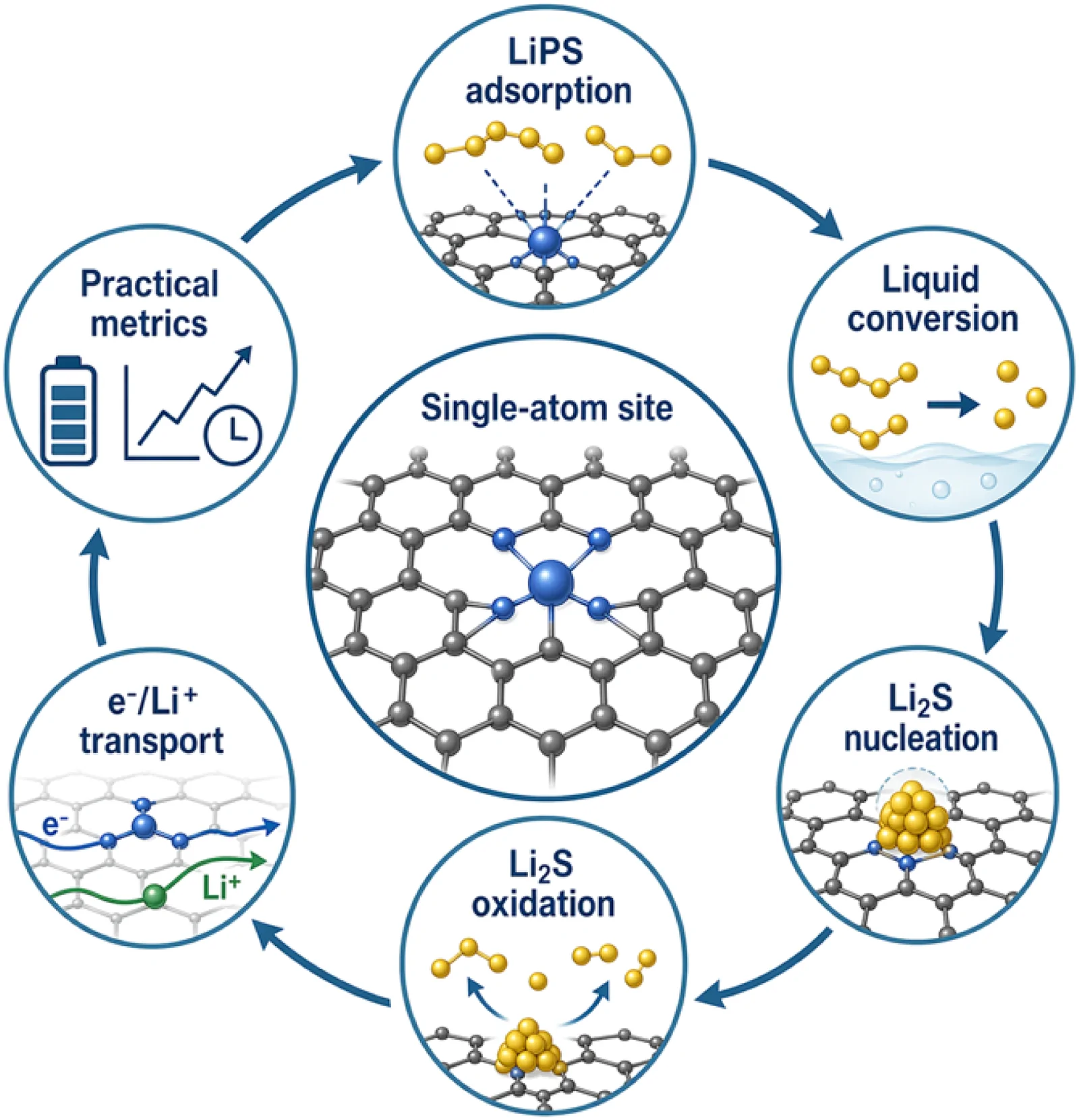
Conceptual circle diagram for SAC-enabled Li–S chemistry. The central single-atom catalytic site on a nanostructured framework is connected to six coupled functions: LiPS adsorption, liquid–liquid conversion, Li_2_S nucleation, Li_2_S oxidation, electron/ion transport, and practical-cell metrics. The circular layout emphasizes feedback between catalyst coverage, framework transport, reaction reversibility, and cell-level reporting. This is an original schematic for this review.

This review therefore uses a mechanism-centered framework. It treats SACs as catalytic components in a multiphase electrochemical reactor rather than as a simple material class. The discussion connects metal identity, coordination environment, framework topology, and interface structure to LiPS adsorption, sulfur reduction, Li_2_S nucleation, Li_2_S decomposition, and full-cell performance. Fig. 1 summarizes the logic used throughout the review. The scope is deliberately selective. It does not treat every sulfur host, separator coating, or lithium-protection layer as equivalent to a SAC strategy. Instead, it focuses on systems where atomically dispersed metal centers are the stated catalytic component or where related atomically precise catalysts provide mechanistic baselines. Conventional polar hosts and molecular catalysts are included only when they clarify a mechanism that also controls SAC behavior, such as adsorption–diffusion balance, Li_2_S oxidation, or high-loading electrode design. This scope is important because Li–S performance can be improved by many non-SAC factors, including sulfur particle size, electrolyte composition, conductive additive distribution, binder chemistry, cathode porosity, separator wetting, and lithium surface area. Without separating these factors, the contribution of a single-atom site can be overstated. A high capacity in a SAC-containing electrode should therefore be interpreted as evidence of catalyst-framework-electrode cooperation, not as proof that the isolated metal atom alone controls the cell.

For this review, a SAC is defined as an isolated, mononuclear metal center immobilized on a solid framework, with no persistent metal–metal coordination detectable by the available structural evidence. Atomically dispersed catalyst (ADC) is used as an umbrella term that can include SACs, dual-atom catalysts (DACs), subnanometer clusters, and molecular or polyoxometalate sites. DACs, clusters, and molecular complexes are not counted as SACs here; they are included only as explicitly labeled mechanistic comparators when they reveal cooperative binding, relay catalysis, or size effects. Conventional heterogeneous catalysts contain multi-atom ensembles in nanoparticles or extended phases and are used only as framework or kinetic benchmarks. An SAC assignment therefore requires convergent, statistically meaningful microscopy and X-ray absorption evidence rather than the absence of diffraction peaks alone.

The review contains six sections. After this introduction, Section 2 discusses the design logic of SACs and their nanostructured frameworks. Section 3 analyzes catalytic mechanisms in sulfur redox chemistry, including adsorption, conversion, Li_2_S nucleation, and Li_2_S decomposition. Section 4 compares quantitative structure-performance relationships across cathode hosts, separator coatings, interlayers, and high-loading cells. Section 5 evaluates limitations, inconsistent test protocols, and future directions. Section 6 concludes with a balanced assessment of what SACs have demonstrated and what remains unresolved for practical Li–S batteries.

## 2. Design logic of single-atom catalysts anchored on nanostructured frameworks

### 2.1. Metal centers and coordination environments

The catalytic function of a SAC is defined by the metal atom and its first coordination shell. In Li–S batteries, Fe, Co, Ni, Zn, V, Mo, Ti, Mn, Cu, W, and noble-metal centers have all been examined experimentally or computationally.^54–66^ Early single-site studies showed that Co–N–C, Co–N/G, and Co-doped mesoporous carbon can increase LiPS affinity and accelerate sulfur redox kinetics.^54,67,68^ Later work expanded to V, Mo, Ni, Zn, Ti, and asymmetric Co configurations. This expansion reflects a mechanistic requirement: the site must bind LiPSs strongly enough to suppress diffusion, but not so strongly that sulfur intermediates become inactive deposits. It must also weaken the Li–S bond in Li_2_S or Li_2_S_2_ during charge without causing irreversible sulfur poisoning or metal aggregation.

M–N_4_ moieties are the most common models because they are relatively stable, can be produced from nitrogen-rich precursors, and can be described with density functional theory (DFT). However, a fully symmetric M–N_4_ unit is not always optimal. Unsaturated Co–N_2_ sites have been reported to interact more strongly with LiPSs and to promote Li_2_S deposition/dissociation relative to Co–N_4_ sites.^61^ Mixed coordination, such as Co–N_3_PS or Co–O_2_N_2_, changes the electron density around the metal center and can strengthen d–p orbital hybridization with sulfur species.^60,62^ Out-of-plane Pt–S coordination in a sulfurized porous organic polymer shifts the local geometry away from planar M–N_4_ motifs and has been used to create ultrathin separator coatings with low Tafel slopes for LiPS conversion.^59^ These examples show that the coordination shell should be treated as an adjustable catalytic ligand field, not only as an anchoring motif.

Metal identity also controls the balance between LiPS binding and Li_2_S conversion. In a computational and experimental screening of single atoms on N-doped graphene, V showed a lower calculated Li_2_S decomposition barrier than Co, Fe, Mn, Ru, and Zn.^55^ The same study reported that graphene required 2.12 eV for Li_2_S decomposition, whereas V on N-doped graphene required 1.10 eV. Co, Fe, Mn, Ru, and Zn sites fell between these extremes. Such comparisons are useful because they evaluate the charge step, which is often less discussed than discharge adsorption. They also caution against ranking SACs only by adsorption energy. Excessively strong binding may suppress shuttle in a static adsorption test but can slow surface transport and product removal.

Coordination number and local symmetry also influence how a metal center interacts with LiPSs. A metal atom embedded in a planar M–N_4_ pocket is usually stable, but its axial accessibility can be limited if the carbon plane is densely graphitized or blocked by deposits. Lower-coordination structures such as M–N_2_ or asymmetric M-N_*x*_Sy/Pz environments can expose stronger axial binding sites, but they may also be less thermally or electrochemically stable. Mixed coordination can polarize the metal center and tune the distribution of occupied and unoccupied d orbitals. This is relevant because LiPS activation involves both accepting electron density from sulfur species and donating electron density into antibonding orbitals. A descriptor such as d–p hybridization is therefore more chemically informative than simply stating that the host is “polar”. However, such descriptors must be supported by experimental signatures, including XANES edge shifts, EXAFS coordination fitting, and post-cycling changes in metal–ligand scattering.

### 2.2. Framework functions beyond site stabilization

The nanostructured framework is not an inert support. It determines whether the single atom can operate under realistic sulfur loading. Conductive graphene and carbon nanotubes provide electron paths and flexible scaffolds, while porous carbon nanocages, hollow capsules, aerogels, and bacterial-cellulose-derived carbon fibers add space for sulfur and reduce diffusion distances.^55,67–73^ MOF-derived carbons offer atomically dispersed metal-nitrogen motifs and hierarchical pores inherited from the precursor.^74–82^ MXenes and metal compounds provide polar surfaces and high conductivity, although single-atom stabilization on these surfaces can be more difficult.^83–87^ Porous organic polymers can coordinate metal atoms through designed ligands and form thin ion-sieving separator layers.^59,88^

Framework design must reconcile four demands. The first is high sulfur accommodation. The host should allow sulfur loadings above 4–6 mg cm^−2^ without blocking electrolyte access. The second is low inactive mass. A high catalyst loading can improve apparent kinetics but reduce cell-level energy density. The third is continuous transport. Electrons must reach the reaction interface, and Li^+^ must pass through pores or interlayers without excessive tortuosity. The fourth is mechanical and chemical stability. Li_2_S precipitation, sulfur volume change, and electrolyte decomposition can cover or corrode active sites. These functions explain why three-dimensional current collectors and ultrathin separator coatings are often more practical than thick catalytic powders added to sulfur composites.

The same framework may play different roles depending on where it is placed. In a sulfur cathode, the framework must store sulfur and conduct electrons while leaving enough void volume for product formation. In a separator coating, the framework mainly regulates LiPS transport and catalyzes conversion at a membrane-like interface. In an interlayer, the framework can act as a secondary current collector and reaction zone. In a lithium-protection layer, a single-atom or MOF-derived framework may homogenize Li^+^ flux and reduce parasitic polysulfide attack. These locations should not be compared only by specific capacity. A cathode host should be judged by sulfur utilization and areal capacity, whereas a separator should also be judged by coating mass, ionic resistance, self-discharge, and anode morphology. A Janus separator should be judged by both cathode-side LiPS conversion and anode-side lithium stability. This location-specific evaluation is still uneven across the literature.

**Fig. 2:**
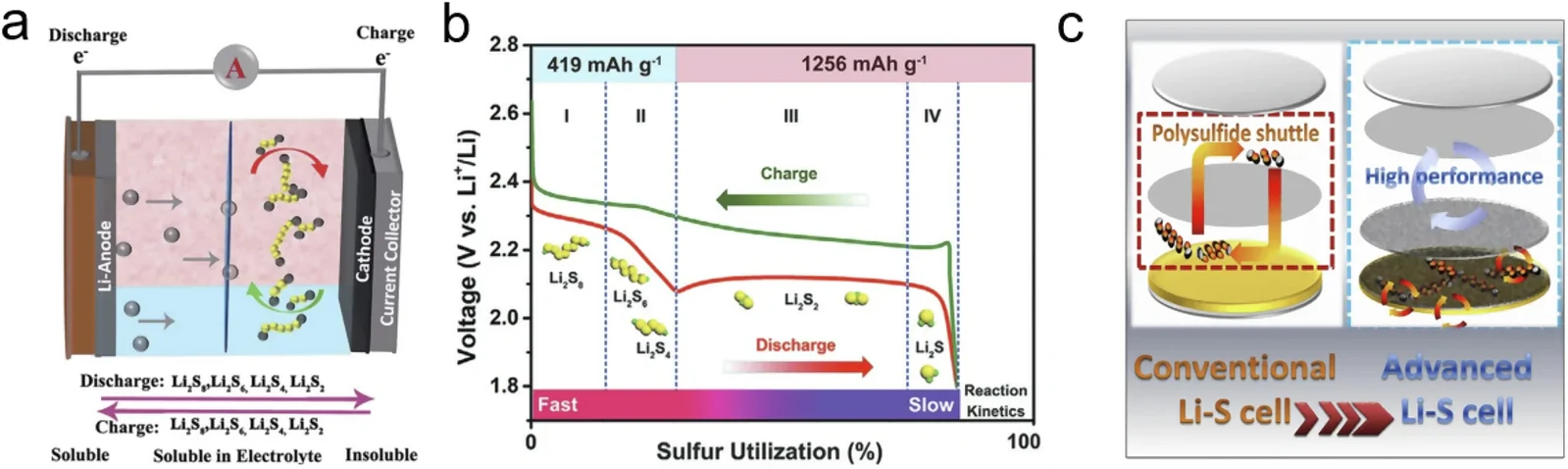
Li–S reaction pathway and shuttle mechanism in a nanostructured cell. (a) An illustration of the charging and discharging process that depicts the generation of polysulfides (Li_2_S_8_, Li_2_S_6_, Li_2_S_4_, and Li_2_S_3_) and insoluble Li_2_S_2_/Li_2_S in a rechargeable Li–S batteries. (b) The standard charging and discharging voltage pattern of Li–S batteries also shows the existence of various intermediate sulfur compounds. (c) Representation of the shuttle mechanism that occurs in a Li–S battery. Adapted from ref. 48 with permission from Springer Nature, copyright 2025.

Fig. 2 places this framework logic in the broader Li–S conversion pathway. It is useful because it distinguishes the sulfur cathode, separator, electrolyte, and lithium anode rather than treating the cell as a single cathode reaction.

### 2.3. Synthetic routes and characterization requirements

Common SAC synthesis routes include pyrolysis of MOFs or zeolitic imidazolate frameworks, impregnation followed by high-temperature coordination, molten-salt-assisted exfoliation/evaporation, atomic layer deposition, chemical vapor deposition, and ligand-directed polymerization.^74–82,88–91^ MOF-derived methods are particularly common because metal ions and nitrogen-containing organic linkers are mixed at the molecular scale before carbonization. Nevertheless, high-temperature pyrolysis can produce metal clusters or nanoparticles if the metal loading, ligand density, and gas atmosphere are not carefully controlled. Acid leaching, secondary annealing, or spatial isolation by Zn evaporation are often used to remove aggregated species or tune the coordination number.^61,76,79^

**Fig. 3:**
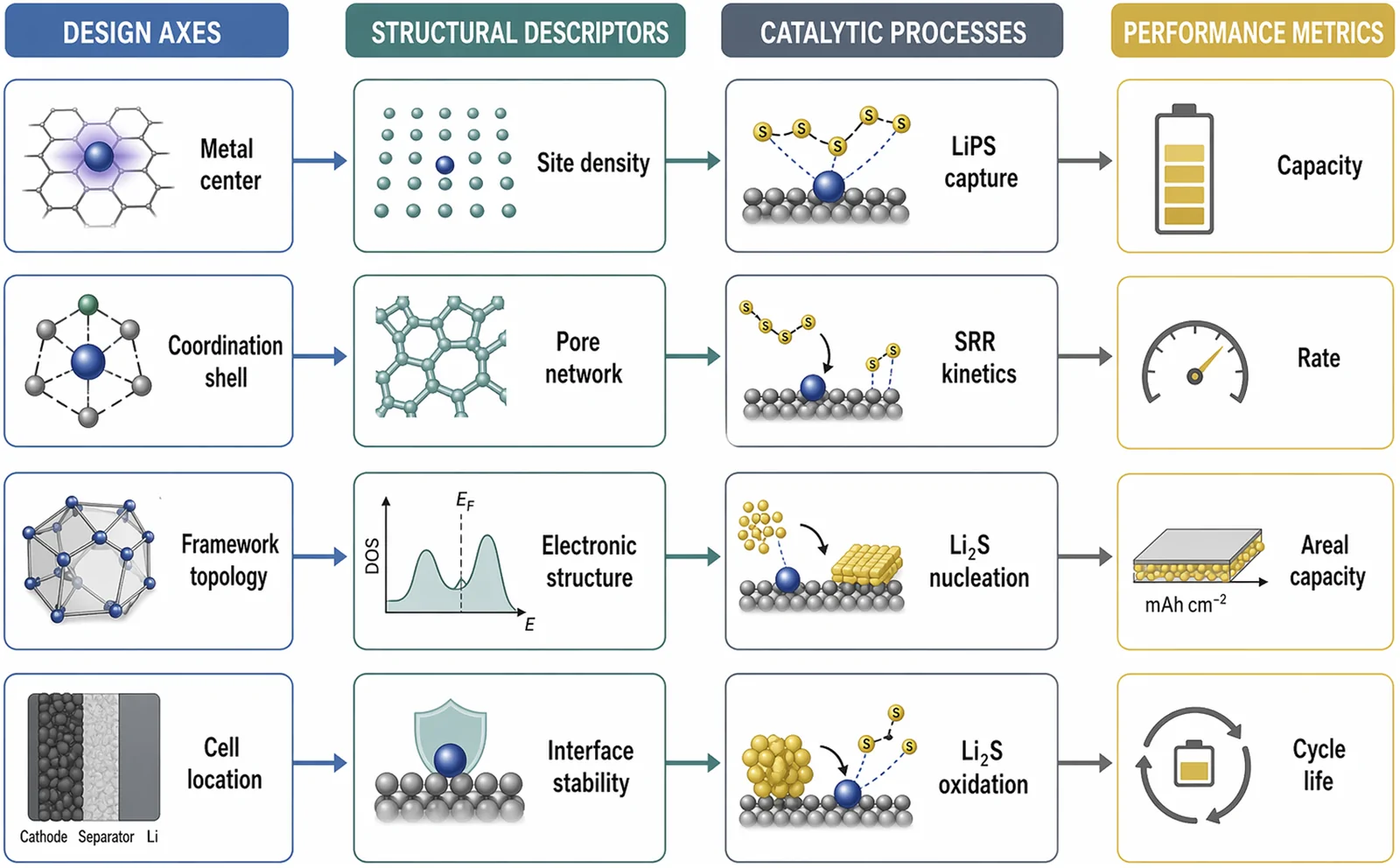
Structure-mechanism map for SAC frameworks in Li–S batteries. The map links four design axes, including metal center, coordination shell, framework topology, and battery location, to structural descriptors, catalytic processes, and cell metrics. The diagram summarizes how SAC design should be interpreted through linked structure, mechanism, and practical test conditions. This is an original schematic for this review.

Pristine-state evidence should be treated as a starting point because an isolated site can reconstruct at each sulfur-conversion state. During cycling, the coordination shell may reorganize; LiPS adsorption may shift metal valence and local electronic structure; partial oxidation or reduction may occur; and Li_2_S_2_/Li_2_S coverage may attenuate or replace the original active interface. A state-resolved protocol should synchronize *operando* XAS with Raman or XRD at matched potentials and times, use cryogenic or quasi-*operando* electron microscopy to preserve soluble/solid intermediates, and test theory-derived structures against the observed spectra. No single technique can separate site reconstruction from changing sulfur-phase fractions, so orthogonal measurements and metal-free, clustered, and pristine-state controls are required (Fig. 3).^37,54,59,71^

Experimental characterization should also cover the catalytic process, not only the catalyst. Symmetric cells with Li_2_S_6_ electrolyte can compare redox currents without full-cell complications, but they do not reproduce the sulfur cathode microstructure. Li_2_S nucleation tests at fixed potential provide information on liquid–solid conversion and deposition capacity, but nucleation current depends on electrode area, electrolyte composition, and wetting. Tafel analysis from cyclic voltammetry can compare kinetic trends, but peak overlap and mass transport can distort slopes. *Operando* XRD, Raman spectroscopy, XAS, ultraviolet-visible spectroscopy, and electron microscopy after defined states of charge are therefore needed to connect site-level design to reaction pathways.^37,39,59,92,93^

The most convincing SAC reports combine structural proof with reaction proof. A typical evidence chain begins with atomically dispersed bright spots in HAADF-STEM, then moves to EXAFS fitting without metal–metal coordination, then demonstrates altered LiPS adsorption or redox currents, and finally connects those effects to full-cell cycling under disclosed conditions. Weak evidence chains usually stop at static adsorption color change or at DFT adsorption energy. Static adsorption is especially limited because it is performed without applied potential, current, product deposition, or lithium metal. DFT adsorption energy is useful when comparing a controlled set of structures, but it is sensitive to model size, solvation treatment, sulfur species, surface coverage, and reference state.

### 2.4. From single active sites to catalytic interfaces

The term “single-atom catalyst” can obscure the fact that the reaction takes place at an extended interface. Once Li_2_S deposits on the catalyst, the original metal center may be partially covered. Wu and co-workers recently showed that Li_2_S crystals grown on a single-atom Ni separator coating can exhibit autocatalytic behavior at the Li_2_S(100)/LiPS interface.^37^ This finding changes the interpretation of catalytic activity. The SAC may be most important at the nucleation stage, while subsequent growth proceeds on the newly formed Li_2_S surface. If this is general, the best catalyst is not necessarily the strongest binder; it is the site and framework combination that creates a favorable Li_2_S orientation, maintains electrolyte access, and avoids electronically isolated deposits.

This concept also explains why conductive and polar frameworks can outperform purely adsorptive materials. Adsorption without lateral diffusion may accumulate LiPSs at dead regions. Conductivity without chemical affinity may allow LiPSs to escape. A useful SAC framework functions as a catalytic relay: soluble LiPSs are captured, converted near an electron path, deposited as accessible Li_2_S, and reoxidized without accumulating thick insulating layers.

Catalytic relay behavior also depends on the distribution of sites inside the electrode. If single atoms are concentrated only on the outer surface of a large host particle, they may intercept LiPSs but leave internal sulfur poorly utilized. If sites are distributed through micropores that are too narrow for solvated LiPS transport, the sites may be inaccessible under realistic current densities. Hierarchical pore design is therefore central. Micropores can confine sulfur and short-chain species, mesopores can accommodate LiPS transport and Li_2_S growth, and macropores can support electrolyte penetration. For SAC hosts, the ideal pore distribution should be matched to sulfur loading and electrolyte amount. A pore network optimized for 1 mg cm^−2^ sulfur in flooded electrolyte may fail at 6 mg cm^−2^ under lean electrolyte because Li_2_S deposition blocks transport paths before the theoretical capacity is reached.

## 3. Catalytic mechanisms in sulfur redox chemistry

### 3.1. Polysulfide adsorption and the adsorption–diffusion balance

LiPS adsorption is the earliest and most visible function of SAC-based materials. Static adsorption tests often show color fading of Li_2_S_6_ or Li_2_S_4_ solutions after adding polar hosts, SAC powders, or separator coatings. Such tests confirm chemical interaction, but they do not prove fast electrochemical conversion. The adsorption interaction can involve metal–sulfur bonding, lithium–heteroatom interaction, Lewis acid–base interaction, or electrostatic confinement in charged frameworks.^35,54,59,76^ For example, Co–N/G binds LiPSs through a Co–N–C center and promotes surface-mediated conversion to and from Li_2_S.^54^ Pt–S–POP/rGO separator coatings combine a porous organic framework with Pt–S coordination, providing both molecular-scale LiPS interaction and a thin ion-sieving layer.^59^

The identity of the adsorbed species is also important. High-order LiPSs such as Li_2_S_8_ and Li_2_S_6_ are more soluble and mobile, whereas low-order species such as Li_2_S_4_, Li_2_S_2_, and Li_2_S are closer to precipitation. A host that strongly binds Li_2_S_8_ may reduce early shuttle but may not accelerate the lower plateau. A host that preferentially stabilizes Li_2_S_2_ or Li_2_S nuclei may improve discharge capacity but can increase charge polarization if product removal is slow. Many papers use Li_2_S_6_ adsorption because Li_2_S_6_ is experimentally convenient and visibly colored. This choice should be stated as a model test, not as a complete description of all sulfur species. Future work should compare multiple polysulfides and, where possible, quantify concentration changes by ultraviolet-visible spectroscopy or chromatography rather than relying only on photographs.

The adsorption-diffusion balance is a useful descriptor for framework design. Tao and co-workers showed that nonconductive oxide surfaces with very strong Li_2_S_*x*_ adsorption can impede diffusion toward conductive carbon, whereas oxides with more balanced interaction support controlled Li_2_S deposition.^35^ This conclusion applies to SACs as well. If an isolated metal site binds LiPSs too strongly, active sulfur can remain trapped at sites that become electronically or ionically blocked. If the interaction is too weak, shuttle dominates. The optimal binding window depends on the framework conductivity, pore geometry, electrolyte, and current density. This is why adsorption energies should be discussed together with diffusion barriers, charge-transfer resistance, and Li_2_S nucleation behavior.

**Fig. 4:**
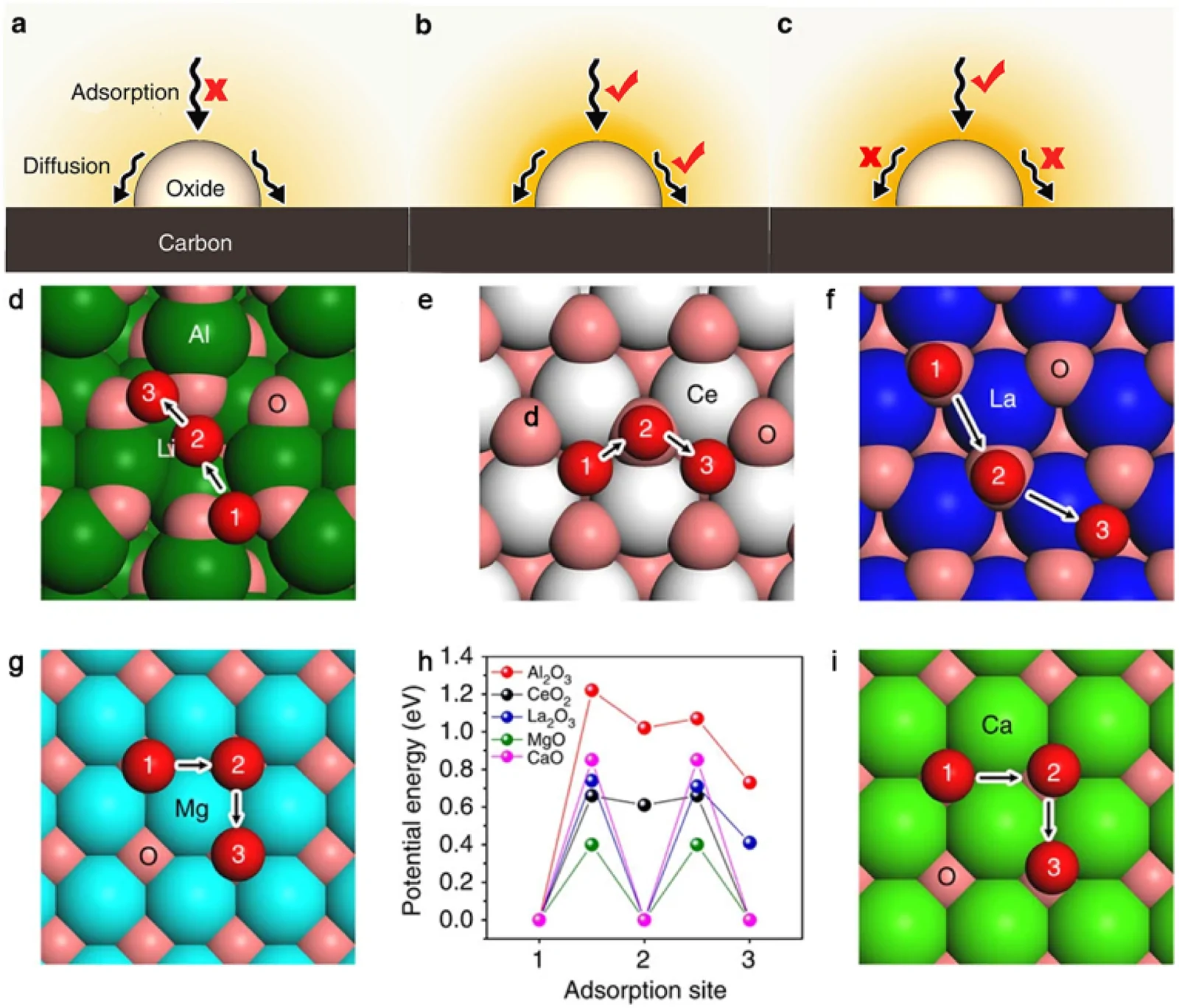
Adsorption and surface-transport balance for LiPS management. (a) The metal oxide with weak Li_2_S_*x*_ adsorption capability; only few Li_2_S_*x*_ can be captured by the oxide; (b) the metal oxide with both strong adsorption and good diffusion, which is favourable for the electrochemical reaction and the controllable deposition of sulfur species; (c) the metal oxide with strong bonding but without good diffusion; the growth of Li_2_S and the electrochemical reaction on the oxide/C surface is impeded. (d–h) Minimum energy path for lithium ion diffusion on Al_2_O_3_(110), CeO_2_(111), La_2_O_3_(001), MgO(100) and CaO(100) surfaces, respectively. (i) Potential energy profiles for Li^+^ diffusion along different adsorption sites on the oxide surface. Adapted from ref. 35 with permission from Springer Nature, copyright 2016.

Solvation adds another layer. LiPSs in ether electrolytes do not approach the catalyst as bare ions; they are coordinated by solvent molecules and paired with Li^+^. LiNO3 and other additives can change the interphase on lithium and modify polysulfide speciation. A DFT model that adsorbs a bare Li_2_S_6_ molecule onto a dry surface can identify trends, but it may overestimate binding compared with solvated conditions. *Operando* spectroscopic data and electrolyte-sensitive simulations are needed to bridge this gap. For engineering analysis, it is safer to interpret calculated adsorption energy as a relative descriptor within one computational series rather than as an absolute predictor of shuttle suppression. Fig. 4 highlights this balance through an oxide model system. Although it is not a SAC figure, it remains mechanistically important because the same adsorption-transport tradeoff appears in single-site hosts.

### 3.2. Sulfur reduction kinetics and liquid–solid conversion

The sulfur reduction reaction in Li–S batteries proceeds through multiple sequential electrochemical transformations rather than a single elementary process. At the high-voltage plateau, cyclo-S_8_ is reduced into soluble long-chain lithium polysulfides, whereas the low-voltage plateau corresponds to their subsequent conversion into insoluble Li_2_S_2_ and Li_2_S.The lower plateau is more sensitive to catalyst design because it requires nucleation and growth of solid products. Peng and co-workers described sulfur reduction as a multistep reaction and showed that the later conversion to insoluble products has larger kinetic resistance than the initial formation of soluble polysulfides.^17^ This mechanistic picture explains why catalysts that improve only the first reduction step can still show poor deep discharge utilization.

Single-atom catalysts can facilitate the liquid–solid conversion process by modulating LiPS adsorption behavior and redistributing the electron density around sulfur sites, thereby reducing the corresponding free-energy barrier. In the study of V anchored on N-doped graphene, the pristine graphene substrate exhibited a rate-determining discharge pathway from Li_2_S_4_ to Li_2_S_2_, with an energy barrier of 1.07 eV. After introducing isolated Co or V sites into N-doped graphene, the kinetically limiting step was transferred to the Li_2_S_2_-to-Li_2_S conversion, and the calculated barriers decreased to 0.72 eV for SACo@NG and 0.84 eV for SAV@NG, respectively.^55^ This comparison shows that a SAC can change both the magnitude and identity of the rate-determining step. It also shows why the lowest Li_2_S decomposition barrier during charge does not automatically imply the lowest discharge barrier. The catalytic site must be assessed in both directions.

**Fig. 5:**
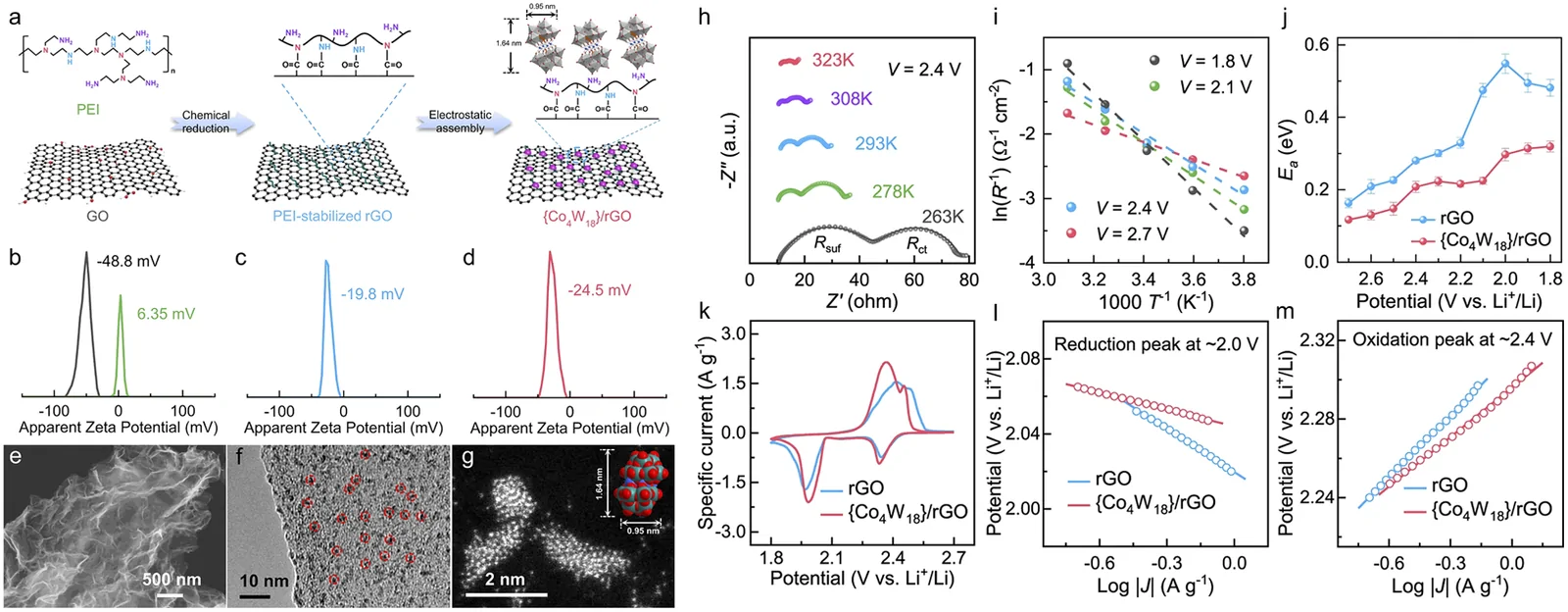
Defined catalytic clusters as a model for bifunctional sulfur electrocatalysis. (a) Synthetic scheme for {Co4W18}/rGO materials. (b–d) Apparent zeta potentials of GO, PEI, PEI-modified rGO, and {Co4W18}/rGO. (e) SEM, (f) HRTEM, and (g) HAADF-STEM images of {Co4W18}/rGO. (h) Temperature-dependent Nyquist plots of {Co4W18}/rGO/S cathodes at 2.4 V. (i) Arrhenius plots based on the relationship between log(1/*R*_ct_) and reciprocal absolute temperature at 2.7, 2.4, 2.1, and 1.8 V. (j) Potential-dependent activation–energy profiles of rGO/S and {Co4W18}/rGO/S cathodes. (k) Fifth-cycle CV curves of rGO/S and {Co4W18}/rGO/S cathodes at 0.1 mV s^−1^. (l and m) Tafel plots derived from the reduction peak at ∼2.0 V and oxidation peak at ∼2.4 V. The polyoxometalate cluster is included as an atomically precise mechanistic comparator and is not classified as a SAC in this review. Adapted from ref. 58 with permission from Springer Nature, copyright 2022.

Polyoxometalate clusters embedded on multilayer graphene provide another example of bifunctional catalytic behavior. Lei and co-workers reported that {Co_4_W_18_}/rGO decreased the maximum activation energy from 0.55 eV for rGO to 0.30 eV, and the same study used Tafel analysis and temperature-dependent impedance to compare sulfur redox kinetics (Fig. 5).^58^ While these polyoxometalate clusters are not isolated single atoms, their molecularly dispersed metal-oxo structures illustrate the value of defined catalytic units on conductive frameworks. They also provide a bridge between molecular catalysts and atomically dispersed metal sites.

### 3.3. Li2S nucleation, growth, and catalyst coverage

Li_2_S nucleation is often used as an operational measure of catalytic activity. In potentiostatic tests, a higher precipitation current, shorter induction time, or larger Li_2_S deposition capacity is interpreted as faster conversion of soluble LiPSs to solid Li_2_S. SACs can promote nucleation by providing polar sites and lowering the interfacial energy of Li_2_S. However, nucleation is not the full reaction. Once Li_2_S covers the catalyst, further growth depends on whether LiPSs can reach the solid–liquid interface, whether electrons can pass through the deposit, and whether the deposit morphology remains accessible.

**Fig. 6:**
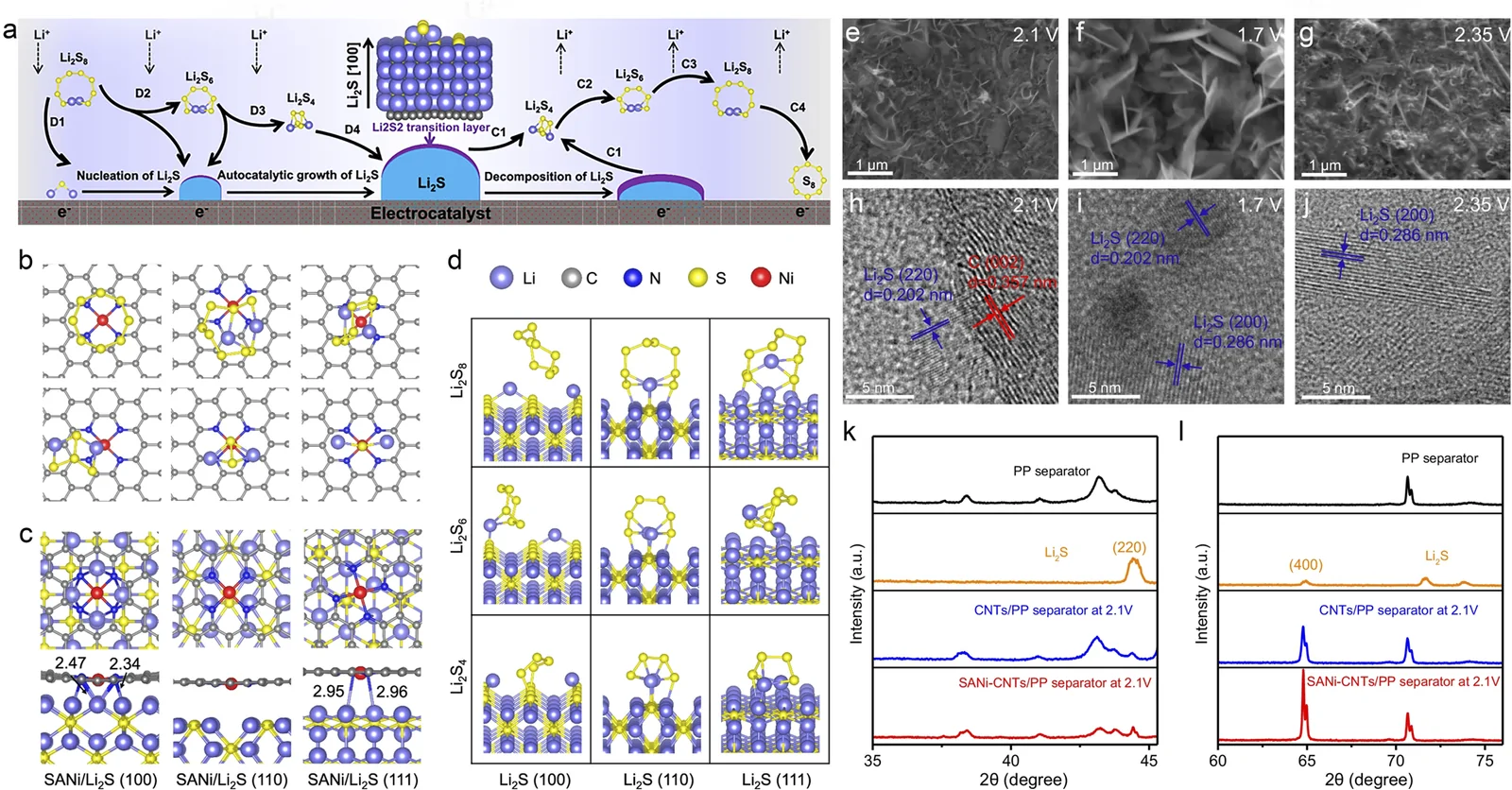
Autocatalytic Li_2_S growth on single-atom Ni separator coatings. (a) Reaction-state sequence from Li_2_S_8_ formation to full discharge (D1–D4) and subsequent recharge to S_8_ generation (C1–C4). (b) Optimized configurations of isolated S_8_ and LiPSs adsorbed on SANi. (c) Optimized interfacial models of SANi with Li_2_S (100), (110), and (111) planes. (d) Optimized adsorption geometries of LiPSs on Li_2_S (100), (110), and (111) facets. (e–g) SEM and (h–j) HRTEM images of Li_2_S nanosheets formed on SANi-CNTs/PP separators at selected discharge/charge states in Li–S cells. (k and l) XRD patterns collected around the Li_2_S (220) and (400) reflections. Adapted from ref. 37 with permission from Springer Nature, copyright 2024.

The SANi study directly addresses this issue. Single-atom Ni on carbon nanotubes was used as a separator coating. DFT calculations showed adsorption energies of −0.95, −1.67, −1.82, −2.38, −2.97, and −3.39 eV for S_8_, Li_2_S_8_, Li_2_S_6_, Li_2_S_4_, Li_2_S_2_, and Li_2_S on SANi (Fig. 6), respectively.^37^ SANi also lowered the Li_2_S_2_-to-Li_2_S free-energy barrier compared with graphene. More importantly, the work proposed that after Li_2_S nucleates on SANi, the Li_2_S(100) surface itself can catalyze further LiPS conversion. The interfacial energy of SANi/Li_2_S(100) was calculated as −0.065 eV Å^−2^, more favorable than the (110) and (111) planes. This explains why Li_2_S nanosheets with preferred orientation can grow and why product coverage does not always stop catalytic conversion.

This mechanism has practical implications. A catalyst that creates small, uniformly distributed Li_2_S nuclei can avoid large inactive agglomerates. A catalyst that produces thick insulating deposits at isolated sites can lose activity rapidly. Separator coatings and interlayers have an advantage in mechanistic studies because they can be designed as well-defined catalytic surfaces without sulfur, binder, and conductive carbon in the same layer. However, their behavior may differ from a thick sulfur cathode, where local electrolyte depletion and pore blockage are more severe.

Li_2_S morphology should therefore be reported together with electrochemical metrics. A high deposition capacity in a potentiostatic test is more meaningful when supported by microscopy showing thin, uniform deposits rather than large blocks. The orientation of Li_2_S crystals, as proposed in the SANi work, may affect both growth and subsequent oxidation.^37^ The same concept can be extended to cathode hosts. If a SAC framework templates Li_2_S as nanosheets or thin films that maintain contact with conductive carbon, charge activation should be easier. If it templates Li_2_S as isolated aggregates in dead pores, the initial nucleation advantage may not translate into cycle life. This is an area where *operando* imaging and post-mortem cross-section analysis can add value.

### 3.4. Li_2_S decomposition and charge kinetics

Charge kinetics are often described through polarization, initial Li_2_S activation voltage, Tafel slope, and calculated Li_2_S decomposition barrier. The PNAS metal sulfide study established that Li_2_S oxidation on catalytic surfaces is a key reaction in Li–S batteries.^36^ In a related single-atom screening study, the calculated energy barriers for Li_2_S decomposition were reported to be 0.30 eV on TiS_2_, 0.31 eV on VS_2_, 0.56 eV on CoS_2_, 0.63 eV on FeS, 1.03 eV on Ni_3_S_2_, and 2.12 eV on graphene.^36,55^ This type of comparison is important because high discharge capacity can be followed by poor reversibility if Li_2_S cannot be oxidized efficiently.

SACs can target charge kinetics through metal-sulfur orbital interactions. In the V-on-N-doped graphene study, the Li–S bond in Li_2_S was weakened by interaction with the single-metal center, lowering the decomposition barrier.^55^ Asymmetric coordination can further tune this effect. Co–N_3_PS, Co–O_2_N_2_, and other mixed-ligand structures have been designed to strengthen orbital hybridization with sulfur species and promote bidirectional conversion.^60,62^ The key is bidirectionality. A site that strongly stabilizes LiPSs during discharge may hinder oxidation if it does not also support Li_2_S delithiation.

Tafel slopes and voltage polarization gaps can serve as experimental indicators of charge-transfer kinetics, although their interpretation requires caution. For Pt-S-POP/rGO separators, the Tafel slopes were measured as 34.6 mV dec^−1^ for the second cathodic peak and 50.2 mV dec^−1^ for the first anodic peak.^59^ These low slopes indicate faster apparent kinetics than pristine PE or less active coatings, but they are extracted from full-cell voltammetry where mass transport and peak overlap can influence values. Tafel analysis is most useful when paired with symmetric-cell redox tests, Li_2_S nucleation, EIS, and post-cycling morphology.

### 3.5. Electronic-structure descriptors

Several descriptors have been proposed for SACs in Li–S batteries. Adsorption energy describes LiPS capture, but it does not fully predict reaction rate. Li_2_S decomposition barrier describes charge activation, but it does not capture discharge nucleation. Gibbs free-energy barriers for sulfur reduction steps describe thermodynamic trends, but they depend on selected reaction pathways. d–p orbital hybridization, d-band center, spin state, and charge density difference can explain why different metal centers or coordination shells change sulfur binding.^44–46,60,62^ Recent computational studies also screen metal centers on model supports such as C_4_N_4_, VS_2_, graphene, and heterointerfaces.^65,66,94–97^

The most useful descriptors are those that combine adsorption and conversion. A volcano-like principle can be expected: weak binding allows shuttle, moderate binding enables capture and conversion, and excessive binding risks site poisoning or slow diffusion. However, the optimum shifts with the framework. A conductive carbon network can tolerate stronger adsorption because electron transfer remains available. A nonconductive or poorly connected framework requires faster surface diffusion. A thin separator coating can use strong LiPS blocking because it does not need to store sulfur, while a cathode host must also maintain pore volume for high sulfur loading.

Descriptor transferability remains limited. A metal center that is optimal on graphene may not be optimal in a MOF-derived carbon, because the local strain, charge distribution, and adjacent heteroatoms differ. A Co–N_4_ site generated in a porphyrinic framework is not necessarily equivalent to a Co–N_4_ site generated by pyrolyzing ZIF precursors. The second coordination sphere, including neighboring N species, vacancies, edges, graphitic domains, and residual oxygen, can change adsorption and charge transfer. For this reason, future computational screening should report not only the metal and first-shell coordination but also the support model, charge state, solvent treatment, and surface coverage. Experimental papers should avoid assigning performance solely to “M–N_4_” unless the coordination environment and site stability have been verified after cycling.

Descriptor uncertainty can be separated into three levels. Model-state uncertainty arises from surface coverage, solvent treatment, Li^+^ coordination, support defects, and the chosen sulfur species. Electrochemical-state uncertainty arises from catalyst oxidation/spin state and the applied potential. Transferability uncertainty arises when absolute energies are compared across different functionals, cells, reference states, or supports. These dependencies can change both the numerical value of a descriptor and the identity of the predicted rate- or potential-determining step.

Table 1 summarizes the physical meaning, strengths, limitations, and experimentally measurable counterparts of the main theoretical descriptors. Potential-dependent studies should, where feasible, examine coverage series, explicit or hybrid solvation, multiple oxidation/spin states, and constant-potential or grand-canonical models. Because these refinements carry different computational costs, absolute values should be compared only within a methodologically consistent series, and conclusions should be supported by sensitivity analysis rather than a single optimized structure.^35–37,44,55,60,62,65,67^

**Table 1:** Scope, strengths, limitations, and experimental counterparts of theoretical descriptors used for SAC catalysis in Li–S batteries

Descriptor	Physical meaning	Principal strength	Main limitations/confounders	Experimental counterpart (not necessarily one-to-one)	Representative refs.
Adsorption energy (Δ*E*_ads_)	Relative binding of LiPSs or solid products to a model site	Rapid screening of trapping trends and binding windows	Strongly dependent on sulfur species, surface coverage, solvent/Li^+^ coordination, oxidation state, potential, and reference state; does not measure conversion rate	Adsorption isotherms or UV-vis depletion, XPS/XANES shifts, and desorption trends	35, 55 and 67
Reaction free-energy profile (Δ*G*)	Thermodynamic driving forces and potential-determining steps along an assumed pathway	Compares multistep sulfur reduction and oxidation within one model series	Depends on pathway/speciation, solvation, entropy corrections, product coverage, and electrode potential; the largest uphill step is not automatically the kinetic barrier	Voltage profiles and *operando* sulfur-speciation trends	17, 37 and 55
Transition-state or NEB barrier (*E*_a_)	Activation energy for a specified bond-breaking, diffusion, or conversion event	Separates kinetic resistance from reaction thermodynamics	Requires a defensible pathway and endpoints; barriers change with coverage, solvation, Li transport, and catalyst reconstruction	Temperature-dependent EIS/current, apparent activation energy, and Tafel analysis used cautiously	36, 55 and 58
d-band center, PDOS, and d–p hybridization	Energy alignment and orbital coupling between metal d states and sulfur p states	Explains coordination- and support-dependent activity trends	A scalar d-band value can hide orbital symmetry, spin, occupancy, and support effects; correlation does not establish the rate-determining step	XAS/XES, UPS/XPS valence spectra, and ligand-field-sensitive spectroscopy	44, 60, 62 and 66
Bader charge or charge-density difference	Partitioned electron redistribution on adsorption or coordination	Visualizes polarization and possible donor/acceptor interactions	Partitioning-scheme and model dependent; charge magnitude is not a direct activity metric	XPS/XANES chemical shifts, work-function/Kelvin-probe changes	44, 60 and 62
Spin state or magnetic moment	Orbital occupancy and exchange state of the metal center	Connects ligand field to bonding and activation of sulfur species	Sensitive to functional, oxidation state, adsorbate, and geometry; competing spin states may interchange during cycling	XES, EPR, magnetic measurements, and spin-sensitive XAS	44, 60 and 65
Work function or constant-potential energetics	Electron chemical potential and reaction energetics under applied bias	Moves the model closer to electrochemical operating conditions	Sensitive to charge compensation, cell size, solvent/counterions, and potential reference; computationally demanding	Open-circuit/polarization data, *operando* potential, and Kelvin-probe measurements	55 and 67
Interfacial/nucleation energy and diffusion barrier	Facet selection, Li_2_S nucleation/growth, and surface transport	Links a site to product morphology and catalyst coverage	Depends on facet, coverage, strain, conductivity, and idealized interface construction; favorable nucleation can still yield insulating deposits	Potentiostatic nucleation, microscopy, XRD texture, and deposit-thickness analysis	35 and 37

### 3.6. Synergy between single atoms and frameworks

**Fig. 7:**
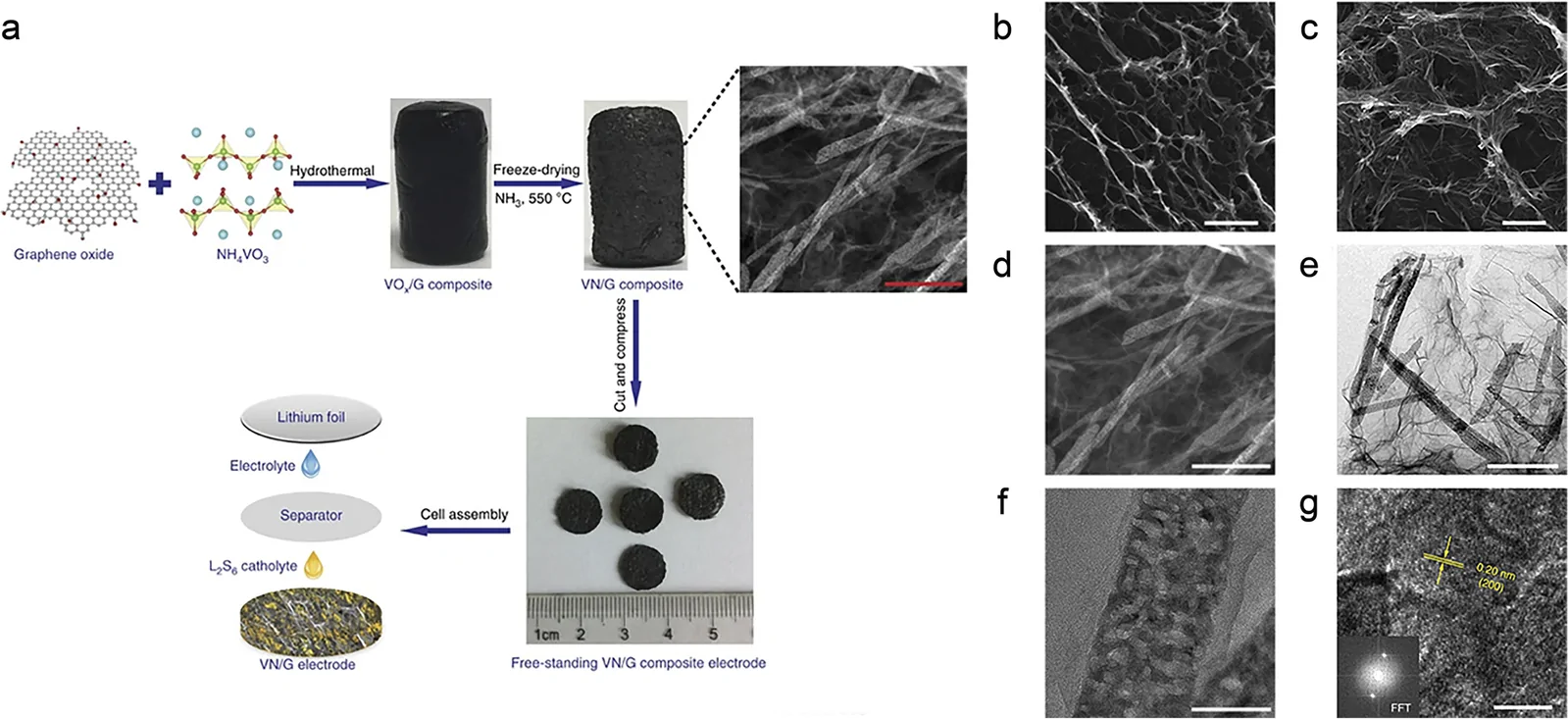
Conductive polar nanostructures as framework analogues for SAC hosts. (a) Fabrication pathway of the porous VN/G composite and corresponding cell assembly, with optical photographs of the obtained material. (b) Low-magnification SEM, (c) high-magnification SEM, (d) HAADF-STEM, and (e and f) TEM images of the porous VN/G composite. (g) HRTEM image with the corresponding FFT pattern shown in the inset. Adapted from ref. 33 with permission from Springer Nature, copyright 2017.

The single atom and the framework act together. In Co–N/G, the graphene matrix supplies conductivity and the Co–N–C center catalyzes reversible LiPS/Li_2_S conversion.^54^ In SAV@NG, the N-doped graphene matrix anchors isolated V atoms and simultaneously preserves a large accessible surface area together with well-dispersed sulfur species.^55^ In MOF-derived catalysts, the precursor can generate uniform metal-nitrogen sites, porous carbon, and sometimes hollow or cage-like morphology.^74–82^ In Pt-S-POP/rGO, a molecularly designed POP coordinates Pt and rGO forms a thin, mechanically stable separator coating.^59^ These examples suggest that “metal center ranking” without framework context is incomplete. Fig. 7 emphasizes nanostructure and conductivity using a VN/graphene host, which is not a single-atom material but remains a useful comparison for framework design. It shows how a polar conductive component and graphene can create void space, electron paths, and adsorption sites.

The catalytic site density must also be optimized. Too few sites may be insufficient for high sulfur loading; too many metal atoms can aggregate or add inactive mass. Very high metal content in a “single-atom” material should be supported by quantitative XAS, microscopy statistics, and post-cycling analysis. In practical electrodes, the amount of catalyst per sulfur mass and the amount of coating per separator area should be reported. The Pt-S-POP/rGO separator is particularly noteworthy because its coating introduced only a minimal inactive component, with a mass loading of approximately 0.04 mg cm^−2^ and a thickness below 150–160 nm, while still enhancing rate capability and cycling stability.^59^ This type of thin catalytic layer is more relevant to cell-level energy density than thick coatings with excellent laboratory cycling but high inactive mass.

## 4. Quantitative structure-performance relationships

### 4.1. Cathode-host SACs

Cathode-host SACs integrate the catalytic site with the sulfur reservoir. Their advantage is proximity: sulfur, LiPSs, Li_2_S, and active sites coexist in the same porous scaffold. Their challenge is coverage and pore blockage. Table 2 compares representative SAC cathode hosts and related atomically dispersed frameworks. The table reports values only when they are explicitly stated in the cited study. NR denotes not reported; no value is inferred from plots or analogous cells.

**Table 2:** Quantitative comparison of representative SAC cathode hosts and related atomically dispersed sulfur hosts

Catalyst/framework	Metal/coordination	Framework role	Sulfur loading or content	Rate/current	Reported capacity/cycling metric	Areal capacity	*E*/*S* ratio	Main mechanistic point	Ref.
S@Co–N/G	Co–N–C	N-doped graphene host	90 wt% S; 6.0 mg cm^−2^	0.2C	1210 mAh g^−1^; 0.029% decay per cycle over 100 cycles	5.1 mAh cm^−2^	NR	Bifunctional Li_2_S formation/decomposition	54
S-SAV@NG	V–N–C	N-doped graphene host	80 wt% S; 5 mg cm^−2^ high-loading test	3C; 0.2–2 C high-loading rates	645 mAh g^−1^ at 3C; high-loading 1143 mAh g^−1^ at 0.2C	NR	NR	Low Li_2_S decomposition barrier and fast LiPS conversion	55
S-SAV@NG long-cycle cell	V–N–C	N-doped graphene host	5 mg cm^−2^	0.5C	645 mAh g^−1^ initial, about 485 mAh g^−1^ after 200 cycles	NR	NR	High-loading cycling with single V sites	55
S-SACo@NG	Co–N–C	N-doped graphene host	NR	0.2C; 0.5C	675 mAh g^−1^ after 100 cycles; 749 to 513 mAh g^−1^ over 400 cycles	NR	NR	Co sites improve LiPS redox compared with N-doped graphene	55
Atomic Co in mesoporous carbon	Co dopant	Mesoporous carbon sulfur host	NR	0.5C	1130 mAh g^−1^; 74.1% retention after 300 cycles	NR	NR	Atomic Co improves LiPS affinity and redox kinetics	67
Co–N/G high-S composite	Co–N–C	Graphene host	90 wt% S	NR	High gravimetric capacity reported with *operando* XAS support	NR	NR	Co–N–C as surface-mediated conversion center	54
Fe single-atom hollow mesoporous carbon/CoS_*x*_-CC	Fe single atoms plus CoSx	Carbon cloth supported composite host	NR	0.1C; 2C; 1C cycling	1430 mAh g^−1^ at 0.1C; 588 mAh g^−1^ at 2C; 0.029% decay per cycle over 600 cycles at 1C	NR	NR	Combines Fe single atoms with polar sulfide current collector	72
FeSA-NC@CBC	Fe–N–C	Bacterial-cellulose-derived carbon nanofiber aerogel	2.5 and 5.0 mg cm^−2^	2C; 1C; 0.5C	840 mAh g^−1^ at 2C; 799.8 mAh g^−1^ at the 500th cycle at 1C; 861 mAh g^−1^ at the 100th cycle at 0.5C under 5.0 mg cm^−2^	NR	NR	MOF-derived Fe nanocages accelerate Li_2_S/Li_2_S_2_ nucleation	77
Ni–N_5_/HNPC	Ni–N_5_	Hollow porous carbon capsules	5.1 mg cm^−2^ high-loading test; 1.3–1.6 mg cm^−2^ standard test	0.2C; 4C; 0.5C cycling	1188 mAh g^−1^ initial at 0.2C; 684 mAh g^−1^ at 4C; 798 mAh g^−1^ after 500 cycles at 0.5C; 0.053% decay per cycle	5.6 mAh cm^−2^ initial and about 4.0 mAh cm^−2^ after 80 cycles at 5.1 mg cm^−2^	NR	Ni–N_5_ coordination balances LiPS adsorption and redox kinetics	78
Ni-NC(p)	Single Ni atoms	Mesoporous N-rich carbon	NR	NR	NR	NR	NR	Porous N-rich host with high sulfur accommodation	79
ZnN_4_-NC	Zn–N_4_	Ordered N-doped carbon nanotube array	7.2 mg cm^−2^	0.1C pouch-cell test; high-loading cycling	5.6 mAh cm^−2^ after 100 cycles under 7.2 mg cm^−2^; pouch cell retained 953.4 mAh g^−1^ after 100 cycles at 0.1C	5.6 mAh cm^−2^	About 3.7 µL mg^−1^	Neighboring Zn single atoms promote fast LiPS conversion at high loading	80
Co–N_2_ graphene	Co–N_2_	N-doped graphene from ZIF-L	7.0 mg cm^−2^ high-loading test	5C; long cycling	687 mAh g^−1^ at 5C; 0.05% decay per cycle for 700 cycles	8.2 mAh cm^−2^	NR	Unsaturated Co–N_2_ improves trapping, deposition, and dissociation	61
CoSA-N3PS	Co–N/P/S	Asymmetric heteroatom-coordinated carbon	6.0 mg cm^−2^ high-loading/pouch-cell test	10C; 5C cycling; 0.2C pouch	619 mAh g^−1^ at 10C; 0.027% decay per cycle after 2000 cycles at 5C; pouch cell retained 660 mAh g^−1^ after 100 cycles at 0.2C	4.4 mAh cm^−2^	NR	Strong d–p orbital hybridization with sulfur species	60
Co–O_2_N_2_ catalyst	Co–O/N	Mixed-coordination porous carbon separator/catalyst	12.0 mg cm^−2^ high-loading test	3C	906.6 mAh g^−1^ at 3C; high-loading areal capacity reached 12.36 mAh cm^−2^	12.36 mAh cm^−2^	NR	Facilitates sulfur species capture and bidirectional redox	62
Ti single-atom 3D electrode	Ti–N–C	Flexible 3D electrode	8 mg cm^−2^; 1 wt% catalyst loading	NR	High sulfur utilization at high area-sulfur loading reported	NR	NR	d–p orbital descriptor favors early transition metals	44
Mn single-atom 3D electrode	Mn–N–C	3D conductive electrode	NR	NR	Comparative activity lower than Ti in cited study	NR	NR	Metal identity changes antibonding occupation	44
Cu single-atom 3D electrode	Cu–N–C	3D conductive electrode	NR	NR	Comparative activity lower than Ti in cited study	NR	NR	Late transition metal has weaker LiPS activation	44
Mo–N_2_/C nanosheets	Mo–N_2_	Atomically dispersed Mo on carbon nanosheets	NR	5C; 2C cycling	743.9 mAh g^−1^ at 5C; 0.018% decay per cycle after 550 cycles at 2C	NR	NR	Promotes Li_2_S deposition/decomposition	68
Fe–N–C/ordered porous carbon	Fe–N_4_	Hierarchically ordered porous carbon	NR	NR	NR	NR	NR	Template-induced trimodal pores and Fe–N_4_ catalytic units	73
Pyridinic-N-rich V SAC	V–N–C	N-rich carbon host	NR	NR	NR	NR	NR	Pyridinic-N coordination boosts LiPS conversion kinetics	64

Cathode-host SACs integrate the catalytic site with the sulfur reservoir. Their advantage is proximity: sulfur, LiPSs, Li_2_S, and active sites coexist in the same porous scaffold. Their challenge is coverage and pore blockage. Table 2 compares representative SAC cathode hosts and related atomically dispersed frameworks. The table gives reported values only where they could be traced from accessible article text, abstracts, or source records. NR denotes information not reported in the consulted source, rather than a value inferred from similar cells.

The strongest conclusion from Table 2 is that the most convincing cathode-host reports provide high sulfur content or loading together with rate and cycling metrics. The Co–N/G and SAV@NG studies remain useful benchmarks because they report high sulfur fraction or high sulfur loading, capacity, rate, and mechanistic evidence.^54,55^ Recent studies often highlight coordination-structure engineering, yet key practical testing parameters are not always fully disclosed in abstracts or publicly accessible records. Therefore, performance comparisons across different reports should be made with caution. A capacity of 1200 mAh g^−1^ measured at 0.1C with a sulfur loading of 1 mg cm^−2^ is not directly comparable to 700 mAh g^−1^ obtained at 1C with a sulfur loading of 5 mg cm^−2^; the latter condition may offer greater relevance for evaluating cell-level energy density.

Another point is the difference between sulfur-based capacity and electrode-level capacity. Most Li–S studies express capacity in mAh g^−1^ normalized to sulfur mass. This metric is useful for judging sulfur utilization, but it does not, by itself, provide a complete basis for evaluating practical energy density. A cathode containing 90 wt% sulfur, as in the Co–N/G work, has a much smaller host penalty than a cathode containing large amounts of catalyst and conductive additive.^54^ Conversely, a low-sulfur composite can deliver high sulfur-normalized capacity while providing modest composite-level capacity. Reviews should therefore avoid ranking materials only by the maximum mAh g_S_^−1^ value. Composite mass, areal sulfur loading, cathode thickness, electrolyte amount, and lithium excess should be included whenever the data are available. When they are not available, the omission itself should be treated as a limitation.

### 4.2. Separator coatings and interlayers

Separator and interlayer designs use SACs differently from cathode hosts. They usually do not store most of the sulfur; instead, they intercept LiPSs, catalyze conversion near the separator/cathode interface, regulate Li^+^ flux, and sometimes protect the lithium anode. The inactive-mass penalty can be low if the coating is thin. However, a thick or poorly conductive coating can increase ion resistance and reduce energy density. Table 3 collects separator/interlayer cases and related test modes.

**Table 3:** Quantitative comparison of SAC separator coatings, interlayers, Janus membranes, and reference separators

Separator/interlayer system	Active site or modifier	Coating/loading feature	Sulfur loading/test condition	Rate/current	Reported performance metric	Self-discharge/anode metric	*E*/*S* ratio	Mechanistic role	Ref.
Co-NPC separator coating	CoSA in N-doped porous carbon	Separator coating	6.2 mg cm^−2^ high-loading test	0.2C; 3C; 1C	1295 mAh g^−1^ at 0.2C; 753 mAh g^−1^ at 3C; 601 mAh g^−1^ after 500 cycles at 1C	7.92 mAh cm^−2^ high-loading areal capacity	NR	Fast and durable LiPS conversion	56
Pt-S-POP/rGO separator	Pt–S out-of-plane SAC	About 120–160 nm coating; about 0.04 mg cm^−2^	2–4 mg cm^−2^ class; high-loading >4 mg cm^−2^	0.5C	1266.8 mAh g^−1^ at 0.5C	NR	NR	Low-polarization catalytic separator	59
Pt-S-POP/rGO separator	Pt–S SAC	Same as above	Standard rate test	0.2C	1360 mAh g^−1^	NR	NR	Maintains liquid–solid conversion at low rate	59
Pt-S-POP/rGO separator	Pt–S SAC	Same as above	Standard rate test	0.5C	1146 mAh g^−1^ in rate series	NR	NR	Lower potential gap than PE	59
Pt-S-POP/rGO separator	Pt–S SAC	Same as above	Standard rate test	1C	915 mAh g^−1^	NR	NR	Faster LiPS conversion	59
Pt-S-POP/rGO separator	Pt–S SAC	Same as above	Standard rate test	2C	764 mAh g^−1^	NR	NR	Rate retention from porous catalytic coating	59
Pt-S-POP/rGO separator	Pt–S SAC	Same as above	Standard rate test	4C	586 mAh g^−1^	NR	NR	High-rate retention	59
Pt-S-POP/rGO separator	Pt–S SAC	Ultrathin coating	Long-term cycling	1C	0.030% capacity fading per cycle over 2000 cycles	Smooth Li anode after cycling reported	NR	Suppresses shuttle and interfacial corrosion	59
Pt-S-POP/rGO separator, high S loading	Pt–S SAC	Ultrathin coating	>4 mg cm^−2^	0.5C	1.8 mAh cm^−2^ initial; 86% retained after 100 cycles	Lower shuttle than PE	NR	High-loading ion-sieving/catalytic function	59
Pt-S-POP/rGO lean-electrolyte test	Pt–S SAC	Ultrathin coating	*E*/*S* = 5 µL mg^−1^	2C	525 mAh g^−1^ at 2C	NR	5 µL mg^−1^	Maintains kinetics under electrolyte constraint	59
PE separator reference in Pt-S-POP work	No SAC	Commercial PE	High-loading comparison	0.5C	0.9 mAh cm^−2^ initial; 45% retained after 100 cycles	OCV final 2.20 V after 72 h	NR	Baseline shuttle and self-discharge	59
Pt-S-POP/rGO self-discharge test	Pt–S SAC	Ultrathin coating	OCV hold 72 h	NR	Capacity loss 161.4 mAh g^−1^	OCV final 2.33 V after 72 h	NR	Reduced LiPS migration	59
PE self-discharge reference	No SAC	Commercial PE	OCV hold 72 h	NR	Capacity loss 327.1 mAh g^−1^	OCV final 2.20 V after 72 h	NR	Severe shuttle reference	59
SAZ-AF Janus separator	Zn SAC plus anionic MOF framework	Janus coating on Celgard	Li–Li symmetric test	5 mA cm^−2^, 10 mAh cm^−2^	NR for Li–S cell in consulted source	52 mV polarization; 2800 h stable cycling	NR	Simultaneous shuttle and dendrite regulation	75
Bio-MOF-100/Celgard side of Janus design	Anionic framework	Anode-facing coating	Li–Li symmetric test	5 mA cm^−2^, 10 mAh cm^−2^	NR for Li–S cell	52 mV polarization with long cycling	NR	Homogenizes Li^+^ flux	75
ZIF-8-derived side of Janus design	Zn SAC	Cathode-facing coating	Li–S separator function	NR	NR	NR	NR	Catalyzes LiPS conversion	75
Ni SAC separator modifier	High-loading Ni SAC	Separator coating	NR	NR	NR	NR	NR	*In situ* trapping strategy for high-performance Li–S cells	82
Co single-atom ordered macro-microporous separator	Co SAC	Ordered macro-microporous layer	NR	NR	NR	NR	NR	Catalytic separator with hierarchical pores	74
FeCo bimetallic single-atom separator	Fe/Co atomically dispersed sites	Separator/anode regulating layer	NR	NR	NR	Dendrite inhibition reported	NR	Couples LiPS conversion and Li regulation	83
CoHP-modified CFP interlayer	Co–N_4_ centers in conductive MOF	Carbon-fiber-paper interlayer	Pouch-cell cycling reported	0.1C	NR	NR	NR	Electrostatic potential induced Co–N_4_ activity	81
Co/NOC separator catalyst	Co–O_2_N_2_	Porous carbon coating on separator	12.0 mg cm^−2^ high-loading test	3C	906.6 mAh g^−1^ at 3C; 12.36 mAh cm^−2^ at high sulfur loading	NR	NR	Mixed O/N coordination promotes LiPS capture and bidirectional redox	62

Two conclusions follow from Table 3. First, separator coatings can provide strong apparent improvements with very small mass, as shown by Pt-S-POP/rGO. This is attractive for practical cells because a 0.04 mg cm^−2^ coating contributes much less inactive mass than a thick catalytic interlayer. Second, separator studies should report sulfur loading and *E*/*S* ratio. A separator can look excellent under flooded electrolyte but may add resistance under lean conditions. The most informative studies include self-discharge, lithium anode morphology, high sulfur loading, and lean-electrolyte rate tests together.

Separator coatings also require separate transport analysis. A coating that blocks LiPSs may also slow Li^+^ migration if it is too dense. A coating with high catalytic activity may still cause polarization if the ion pathway is tortuous or poorly wetted. The Pt-S-POP/rGO example is instructive because the coating is thin and porous, while the Janus separator concept adds different functions on the cathode and anode sides.^59,75^ This suggests that separator design should move from single-function blocking layers toward asymmetric transport/catalysis layers. The cathode side should capture and convert LiPSs, while the anode side should homogenize Li^+^ flux and reduce corrosive attack. Such designs need impedance data, transference or diffusion measurements where possible, and post-cycling separator morphology.

### 4.3. Mechanistic metrics and their limits

Table 4 gathers mechanistic descriptors across SACs and benchmark catalytic surfaces. It illustrates why no single descriptor is sufficient. Adsorption energy, free-energy barrier, Li_2_S decomposition barrier, Tafel slope, interfacial energy, and long-cycle fading rate each describe a different part of the reaction network.

**Table 4:** Mechanistic descriptors reported for SACs and benchmark catalytic surfaces in Li–S batteries

System/model	Metric	Reported value	Reaction/process	Interpretation	Ref.
Graphene	Li_2_S decomposition barrier	2.12 eV	Charge-side Li_2_S delithiation	Poor Li_2_S activation on nonpolar carbon	55
SAV@NG	Li_2_S decomposition barrier	1.10 eV	Charge-side Li_2_S delithiation	V single atom lowers oxidation barrier	55
SACo@NG	Li_2_S decomposition barrier	1.76 eV	Charge-side Li_2_S delithiation	Better than graphene but weaker than V in this model	55
SAFe@NG	Li_2_S decomposition barrier	1.66 eV	Charge-side Li_2_S delithiation	Intermediate activity	55
SAMn@NG	Li_2_S decomposition barrier	1.38 eV	Charge-side Li_2_S delithiation	Lower barrier than Co and Fe in this model	55
SARu@NG	Li_2_S decomposition barrier	1.64 eV	Charge-side Li_2_S delithiation	Noble metal not automatically optimal	55
SAZn@NG	Li_2_S decomposition barrier	1.81 eV	Charge-side Li_2_S delithiation	Weak Li_2_S activation relative to V	55
Graphene	Rate-limiting sulfur reduction free energy	1.07 eV	Li_2_S_4_ to Li_2_S_2_	Sluggish lower-plateau conversion	55
SACo@NG	Rate-limiting sulfur reduction free energy	0.72 eV	Li_2_S_2_ to Li_2_S	Co changes and lowers rate-limiting step	55
SAV@NG	Rate-limiting sulfur reduction free energy	0.84 eV	Li_2_S_2_ to Li_2_S	V improves reduction *vs.* graphene	55
Li^+^ diffusion on screened SACs	Diffusion barrier	About 0.23 eV	Surface Li^+^ diffusion	Bond breaking dominates over diffusion in Li_2_S decomposition	55
TiS_2_	Li_2_S decomposition barrier	0.30 eV	Li_2_S oxidation	Strong catalytic sulfide benchmark	36
VS_2_	Li_2_S decomposition barrier	0.31 eV	Li_2_S oxidation	Strong catalytic sulfide benchmark	36
CoS_2_	Li_2_S decomposition barrier	0.56 eV	Li_2_S oxidation	Conductive polar sulfide improves charge kinetics	36
FeS	Li_2_S decomposition barrier	0.63 eV	Li_2_S oxidation	Moderate catalytic sulfide	36
Ni_3_S_2_	Li_2_S decomposition barrier	1.03 eV	Li_2_S oxidation	Higher barrier than TiS_2_/VS_2_	36
SnS_2_	Li_2_S decomposition barrier	0.32 eV calculated; high experimental activation voltage	Li_2_S oxidation	Low intrinsic barrier offset by poor conductivity	36
SANi	Adsorption energy for Li_2_S_8_	−1.67 eV	LiPS adsorption	Stronger LiPS interaction than graphene	37
SANi	Adsorption energy for Li_2_S	−3.39 eV	Discharge product binding	Strong product anchoring can induce oriented nucleation	37
SANi/Li_2_S(100)	Interfacial energy	−0.065 eV Å^−2^	Li_2_S nucleation/growth	Favors Li_2_S(100) orientation and autocatalytic growth	37
SANi	Free-energy barrier for Li_2_S_2_ to Li_2_S	0.79 eV	Discharge lower plateau	Lower than graphene value of 0.90 eV	37
Pt-S-POP/rGO	Tafel slope, second reduction peak	34.6 mV dec^−1^	LiPS reduction	Faster apparent reduction kinetics	59
Pt-S-POP/rGO	Tafel slope, initial oxidation peak	50.2 mV dec^−1^	Li_2_S/LiPS oxidation	Faster charge-side kinetics	59
{Co_4_W_18_}/rGO	Maximum activation energy	0.30 eV	LiPS conversion to Li_2_S_2_/Li_2_S	Lower than rGO value of 0.55 eV	58

The descriptor comparison has three implications. First, a low Li_2_S decomposition barrier is necessary but not sufficient; SnS_2_ shows that poor electronic conductivity can override favorable intrinsic chemistry.^36^ Second, product binding can be beneficial for nucleation but harmful if the product forms thick insulating deposits. Third, Tafel slopes and activation energies should be interpreted with the test geometry. A separator coating, a symmetric cell, and a composite cathode expose different catalytic interfaces.

The values in Table 4 also show why bidirectional catalysis should be tested explicitly. A site that lowers the Li_2_S_2_-to-Li_2_S barrier during discharge may not lower the Li_2_S-to-LiPS barrier during charge. Conversely, a site that activates Li_2_S oxidation may bind high-order LiPSs weakly and allow shuttle before precipitation. The best SACs should be compared through paired discharge and charge metrics: reduction peak current and oxidation peak current, Li_2_S nucleation capacity and Li_2_S activation voltage, symmetric-cell redox current in both directions, and DFT free-energy profiles for both sulfur reduction and Li_2_S decomposition. Reporting only one side of the cycle can hide kinetic asymmetry.

### 4.4. High-loading and lean-electrolyte relevance

For practical Li–S cells, the key performance descriptors should include areal capacity, sulfur areal loading, electrolyte-to-sulfur ratio, total electrolyte usage, lithium excess, and cycling durability under application-relevant current densities. Capacity normalized only to sulfur mass may overstate performance when the sulfur loading is limited or when the conductive host contributes substantial inactive weight. For instance, a cathode showing 1000 mAh g^−1^ at 1 mg cm^−2^ sulfur delivers an areal capacity of merely 1 mAh cm^−2^, which remains below the level required for many practical designs. By contrast, a cell with a lower sulfur-normalized capacity at 6 mg cm^−2^ may offer greater practical significance, provided that polarization is suppressed and the electrolyte inventory is properly managed.

Practical translation requires a mass and inventory balance rather than sulfur-normalized capacity alone. Catalyst mass fraction and separator-coating mass should be included in electrode- and cell-level specific energy; compatibility with low electrolyte volume should be demonstrated without relying on excess wetting; lithium thickness or the negative/positive capacity ratio should be reported; synthesis yield and batch-to-batch variation should be established; and pouch-cell tests should disclose stack pressure, current collectors/tabs, electrolyte-filling protocol, and failure mode. A SAC architecture should be considered engineering-relevant only when its advantage persists as these constraints are imposed sequentially.

**Fig. 8:**
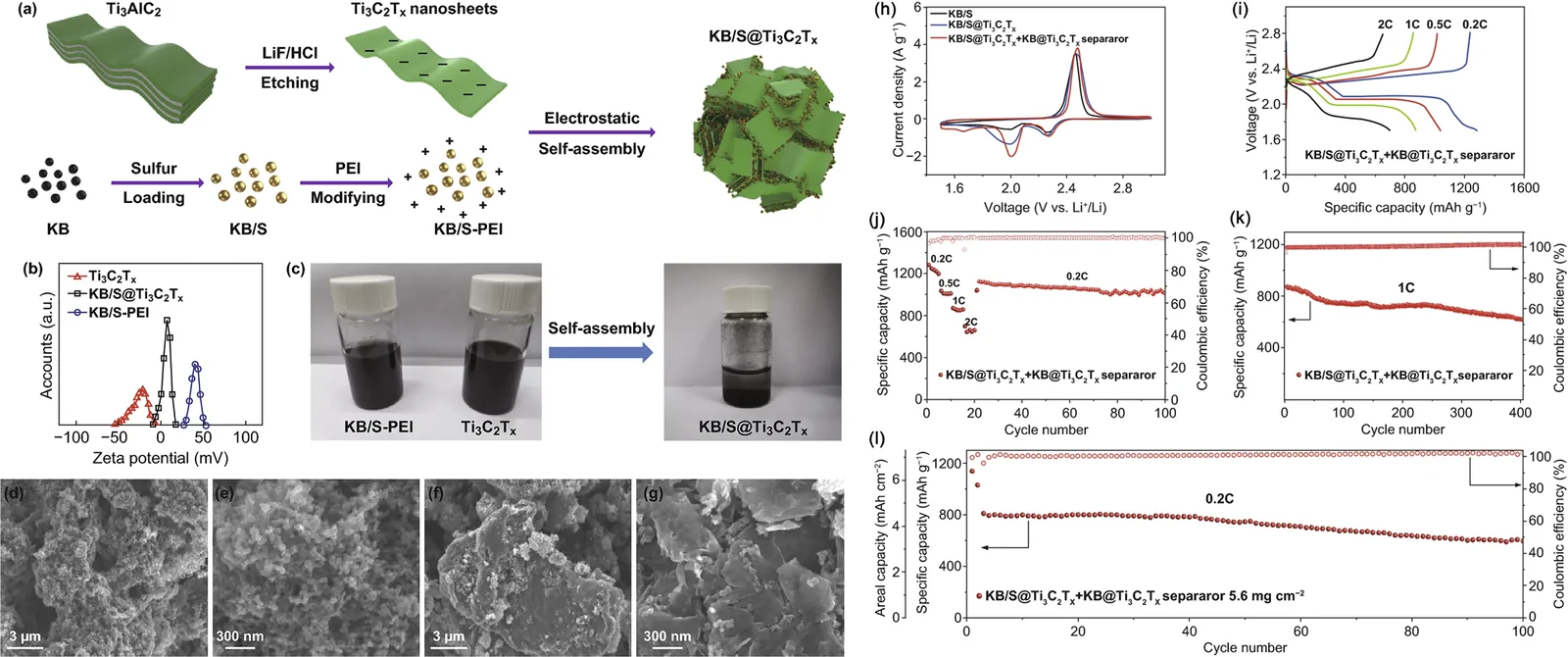
High-sulfur-loading cathode architecture and practical metrics. (a) Fabrication scheme of the KB/S@Ti_3_C_2_T_*x*_ composite. (b) Zeta potentials of Ti_3_C_2_T_*x*_ nanosheets, KB/S-PEI, and KB/S@Ti_3_C_2_T_*x*_. (c) Digital images of aqueous suspensions containing KB/S-PEI, Ti_3_C_2_T_*x*_, and KB/S@Ti_3_C_2_T_*x*_. (d and e) SEM images of KB/S and (f and g) KB/S@Ti_3_C_2_T_*x*_. (h) CV curves of KB/S and KB/S@Ti_3_C_2_T_*x*_ with pristine separators, together with the KB/S@Ti_3_C_2_T_*x*_ electrode coupled with the KB@Ti_3_C_2_T_*x*_-modified separator. (i) Rate-dependent charge/discharge profiles of the KB/S@Ti_3_C_2_T_*x*_ cell using the KB@Ti_3_C_2_T_*x*_-modified separator from 0.2 to 2C. (j) Rate capability and (k) long-term cycling stability at 1C for the KB/S@Ti_3_C_2_T_*x*_ electrode paired with the KB@Ti_3_C_2_T_*x*_ separator. (l) Cycling behavior at a high sulfur loading of 5.6 mg cm^−2^ under 0.2C. Adapted from ref. 87 with permission from Wiley, copyright 2023.

The Co–N/G report represents an early case in which high sulfur content was evaluated together with areal-level performance, showing 90 wt% sulfur, a sulfur loading of 6.0 mg cm^−2^, an areal capacity of 5.1 mAh cm^−2^, and a per-cycle capacity loss of 0.029% over 100 cycles.^54^ In the SAV@NG system, the sulfur loading was increased to 5 mg cm^−2^, while a capacity of 1143 mAh g^−1^ at 0.2C and favorable rate performance under high-loading conditions were achieved.^55^ For Pt-S-POP/rGO modified separators, testing under a lean electrolyte condition of *E*/*S* = 5 µL mg^−1^ gave 525 mAh g^−1^ at 2C, providing a more practically relevant assessment than cycling results obtained only with excess electrolyte.^59^ Co-NPC separator coatings further delivered an areal capacity of 7.92 mAh cm^−2^ at a sulfur loading of 6.2 mg cm^−2^.^56^ These examples should be weighed more heavily than low-loading results without *E*/*S* disclosure. Fig. 8 focuses on high-loading architecture rather than single-atom chemistry. It provides a practical counterpoint: framework engineering and electrolyte management can dominate performance even when the catalyst is active.

### 4.5. Why literature comparisons remain difficult

Quantitative comparison across SAC reports is limited by inconsistent protocols. Different papers use different sulfur loadings, cathode compositions, electrolyte salts, LiNO_3_ concentrations, separators, electrolyte amounts, voltage windows, activation cycles, and current definitions. Some report capacity based on sulfur mass, others on composite mass. Some use high catalyst loading but do not include catalyst mass in energy calculations. Many do not report *E*/*S* ratio or lithium thickness. These omissions can exaggerate the apparent advantage of a catalytic material.

Coin cells also overestimate practical stability. They often use excess electrolyte and lithium foil, which can buffer shuttle and accommodate side reactions. Pouch cells, lean electrolyte, and limited lithium expose problems that coin cells hide, including electrolyte depletion, gas formation, pressure change, lithium corrosion, and nonuniform current distribution.^6,7,40^ SACs can help by accelerating conversion and reducing soluble LiPS concentration, but they cannot compensate for poor electrolyte design, unstable lithium interfaces, or excessive inactive material.

**Fig. 9:**
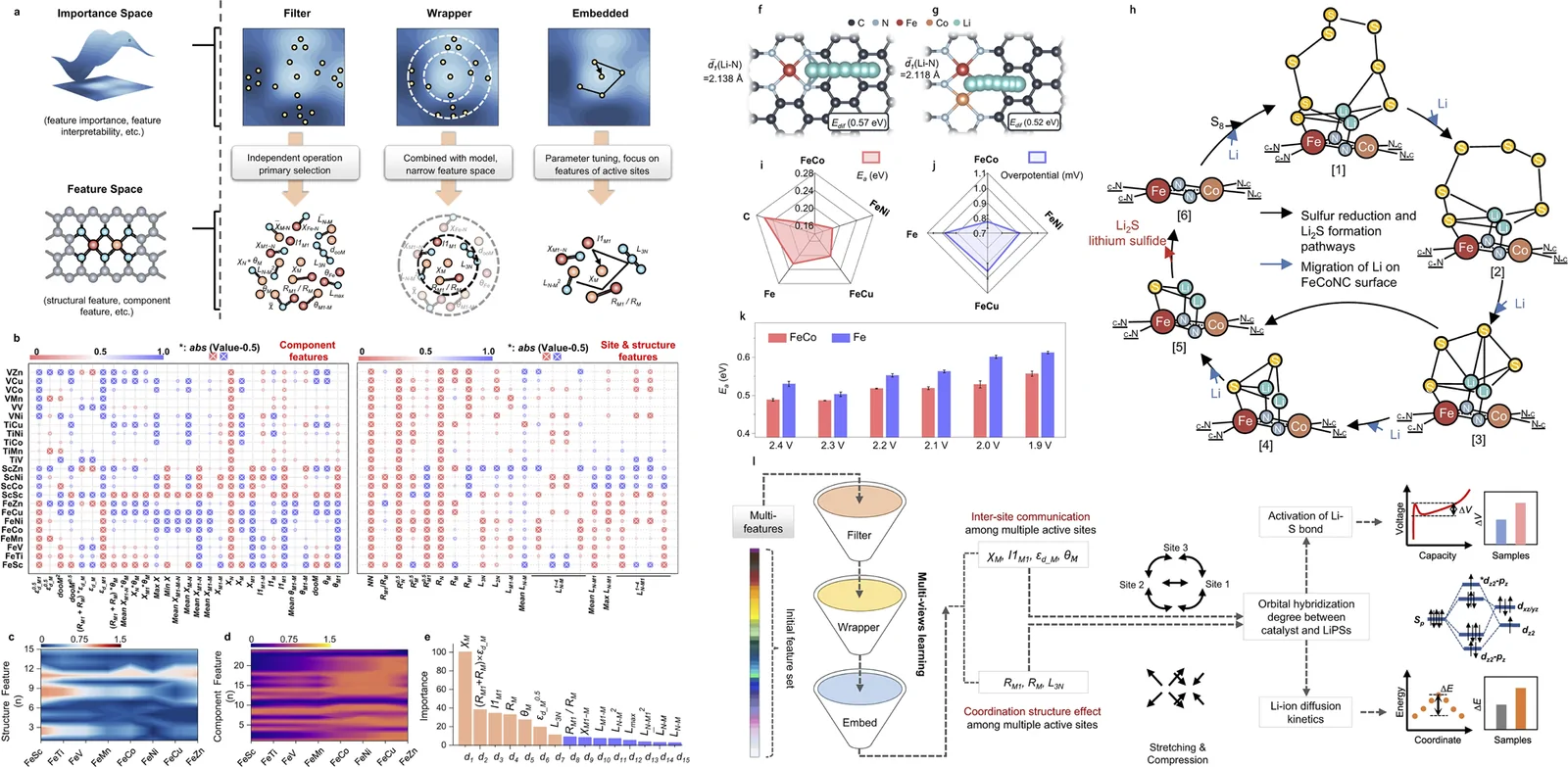
Descriptor-guided catalyst selection for high-energy Li–S cells. (a) Comparative schemes of feature-engineering strategies: filter-based ranking by correlation analysis before model construction, wrapper-based selection through iterative training with a base algorithm, and embedded selection according to feature contribution during model fitting. M1 denotes the primary metal center, M denotes the secondary cooperative metal center, and N denotes the coordinating nitrogen atom. (b) Normalized representation of the initial dataset. (c and d) Feature heat maps for selected DASC systems, where the horizontal axis lists abbreviated DASC labels, the vertical axis gives the feature number, and color intensity reflects the relative feature value. (e) Feature-importance distribution of the final XGBR model obtained from the embedded strategy. Top-view diffusion-pathway models for (f) Fe SASC and (g) FeCo DASC. (h) Proposed sulfur electrochemical conversion mechanism on FeCo DASC driven by ensemble effects. (i) Apparent activation energies for Li_2_S-to-Li_2_S_*n*_ conversion over Fe SASC, FeCo DASC, FeNi DASC, FeCu DASC, and the catalyst-free reference, derived from CV measurements. (j) Overpotentials extracted from CV curves of symmetric cells using Li_2_S_6_ electrolyte, with error bars calculated from standard deviations. (k) Potential-dependent *E*_a_ values at a fixed discharge voltage for Fe SASC and FeCo SASC. (l) Workflow for identifying ensemble effects through a multi-view machine-learning framework. FeCo DASC and the other dual-atom configurations are treated as ADC comparators outside the mononuclear SAC category. Adapted from ref. 96 with permission from ACS, copyright 2021.

For a fair comparison, a minimum reporting set should be adopted. At the electrode level, authors should disclose sulfur loading, sulfur fraction, cathode areal mass, conductive additive and binder fractions, catalyst loading, electrode thickness, porosity if measured, and current collector type. At the cell level, they should disclose electrolyte composition and volume, *E*/*S* ratio, lithium thickness, separator type and coating mass, voltage window, formation cycles, and temperature. At the performance level, they should report specific capacity, areal capacity, retention or decay rate, coulombic efficiency, rate sequence, and the basis used for capacity normalization. At the mechanism level, they should include controls without the metal atom and controls with the same framework but different coordination or aggregation state. These items are not excessive; they are necessary to decide whether a SAC improves practical Li–S chemistry. Fig. 9 is included to connect descriptor-driven material selection with practical high-energy cell design.

## 5. Limitations, standardization, and future directions

### 5.1. Low sulfur loading and excess electrolyte

Many SAC papers still rely on low sulfur loading and high electrolyte amount. Under such conditions, LiPS concentration is lower, ion transport is easier, and cathode flooding can mask poor pore design. A catalyst may appear effective because the cell is not strongly constrained. Practical Li–S cells require high sulfur loading, usually above 4–5 mg cm^−2^, low *E*/*S* ratio, limited lithium excess, and stable areal capacity over many cycles.^5,6,40^ Therefore, future SAC studies should report at least sulfur loading, sulfur fraction in the cathode, electrolyte volume, *E*/*S* ratio, lithium thickness or negative/positive ratio, separator and coating mass, current density, voltage window, and areal capacity.

The comparison should also separate three claims: improved sulfur utilization, reduced shuttle, and faster kinetics. A higher initial capacity may result from better wetting rather than catalysis. A lower capacity fading rate may result from stronger confinement rather than faster conversion. A smaller voltage hysteresis may reflect conductivity improvement rather than a lower intrinsic reaction barrier. Studies should combine control samples that isolate the metal atom, coordination shell, framework, and coating mass. Controls should include metal-free framework, metal nanoparticles or clusters, different coordination environments, and equivalent surface area/conductivity where possible.

### 5.2. Coin-cell bias and the need for practical demonstrations

Coin cells are useful for screening but insufficient for commercialization arguments. The pressure distribution, electrolyte volume, lithium excess, and thermal environment differ from pouch cells. SAC-based cells should be evaluated under staged conditions: standard coin-cell screening, high-loading coin cells with disclosed *E*/*S* ratio, limited–lithium cells, and pouch cells. The literature contains promising examples of high areal capacity and pouch testing, but these remain fewer than conventional low-loading demonstrations.^40,46,81^ The field should avoid describing materials as practical when the evidence is limited to flooded coin cells.

Pouch-cell studies should report stack pressure, *N*/*P* ratio, electrolyte amount, lean-electrolyte wetting protocol, gas generation if measured, and failure mode. A catalyst that performs well in a coin cell may fail in a pouch cell if it increases impedance or if the coating cracks during cycling. Conversely, a modest catalytic improvement may be valuable if it allows lower electrolyte inventory or thinner lithium.

### 5.3. Mechanistic uncertainty under operating conditions

Mechanistic tests are often performed in simplified systems. Symmetric cells use soluble Li_2_S_6_ electrolyte and identical electrodes. Static adsorption tests use dilute LiPS solutions without current flow. DFT models often use isolated molecules, idealized surfaces, vacuum or implicit solvent, and low coverage. These methods are valuable, but their results must be connected to real cathodes where LiPS concentration, electric field, solvent coordination, product coverage, and local pH-like solvation environment vary during cycling.

*Operando* and quasi-*operando* methods are needed. *Operando* XRD can follow Li_2_S and sulfur phases; Raman spectroscopy can track polysulfide species; XAS can monitor metal-site valence and coordination; UV-visible spectroscopy can quantify LiPS diffusion; cryo-TEM and focused-ion-beam cross sections can image deposits after controlled states. The most informative studies combine several methods with electrochemical protocols that match the cell conditions used for performance claims.^33,54,59,92,93,98,99^

Each method resolves a different part of the evolving interface. *Operando* XANES/EXAFS can follow average metal valence and coordination but may miss a minor aggregated population; Raman spectroscopy and XRD resolve sulfur species or crystalline products but do not uniquely identify the active metal site; cryo-TEM preserves deposits at selected states but does not provide continuous kinetics; and *in situ* UV-vis spectroscopy tracks soluble LiPSs but is sensitive to optical path length and cell geometry. Mechanistic assignments are strongest when these signals are correlated with current or capacity at the same potential and electrolyte composition and then compared with explicit candidate structures from theory.

### 5.4. Stability of single atoms

SAC stability is not guaranteed. Atomic deactivation can occur through several non-equivalent routes: reversible coordination or valence reconstruction; irreversible migration and metal–metal aggregation; dissolution or ligand fragmentation followed by crossover to the separator or lithium; and physical burial beneath electronically insulating Li_2_S_2_/Li_2_S. High surface energy and excessive metal loading increase the tendency to coalesce, whereas applied potential, solvated LiPSs, and sulfur-containing intermediates can weaken or reconfigure metal–ligand bonds. These processes can reduce accessible site density, change selectivity, or create cluster activity that is incorrectly attributed to the original SAC.^71^

Stability claims therefore require state-resolved mass and structure balances. *Operando* XANES/EXAFS can track average valence and coordination; quasi-*operando* HAADF-STEM and XPS at matched states can identify irreversible aggregation; ICP-OES or ICP-MS of the electrolyte, separator, and lithium can reveal metal crossover; and post-cycling controls should compare isolated sites, deliberately clustered analogues, and metal-free supports. The recent NiPc/Ni–N–C comparison identifies molecular-site collapse and migration as a distinct failure pathway and shows why immobilization within a conductive carbon coordination environment matters.^71^

Mitigation should prioritize multidentate anchoring, defect or pore confinement, moderate site density, conductive frameworks, and synthesis windows that avoid local metal supersaturation. Stability must be retested at high sulfur loading and low electrolyte inventory because dilute conditions can mask migration, dissolution, and product burial. Scalability should be evaluated in parallel: MOF-derived and polymer-coordinated catalysts can be structurally precise, but precursor cost, solvent use, pyrolysis yield, batch reproducibility, acid-leaching demand, and metal recovery determine whether that precision is practical. Noble-metal SACs may be defensible in ultralow-mass coatings, whereas earth-abundant Fe, Co, Ni, Zn, V, Mn, and Mo sites are more plausible for larger catalyst inventories if their long-term stability and environmental footprint are verified.^67,71^

### 5.5. Future design directions

Among the available deployment modes, the most defensible near-term focus is an ultrathin bifunctional SAC separator/interlayer rather than a high-mass catalytic host. Placing isolated sites at the cathode/separator interface concentrates catalysis where soluble LiPSs cross the cell, allows coating mass and ionic resistance to be controlled independently, and displaces less sulfur than a bulk catalytic host. A cathode-facing porous SAC layer should capture and convert LiPSs, whereas a lithium-facing layer should homogenize Li^+^ flux and limit crossover-induced corrosion. This recommendation is conditional: the coating must remain thin, wettable, mechanically intact, electronically connected, and ionically permeable.^59,70^

**Fig. 10:**
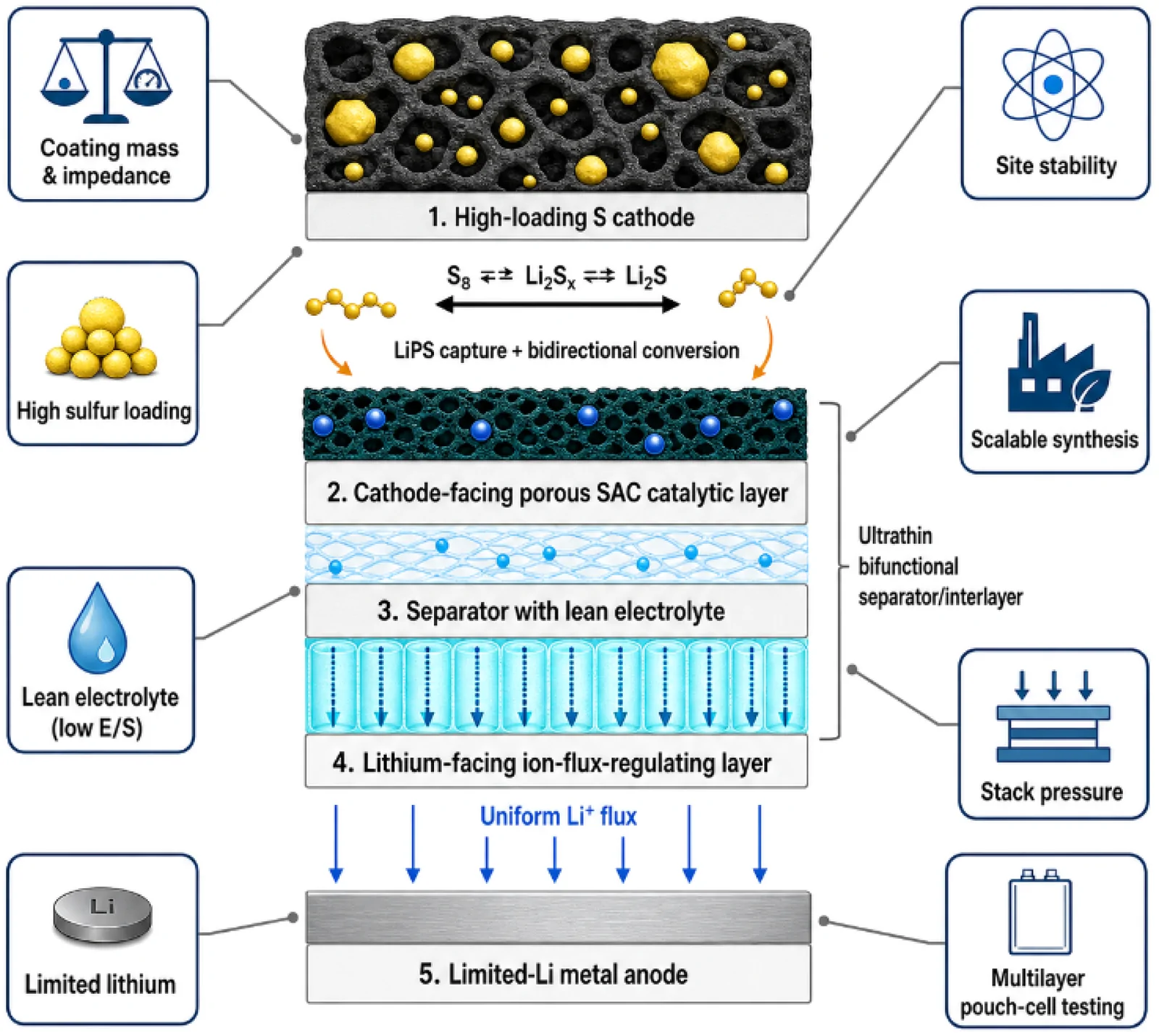
Prioritized translation pathway for SAC-enabled practical Li–S batteries.

A staged evaluation should first report catalyst/coating areal mass, Li^+^ transport resistance, and metal-free and clustered controls; then demonstrate high sulfur loading with the *E*/*S* ratio disclosed; then limit lithium excess; and finally validate multilayer pouch cells with stack pressure and failure mode reported. The Pt-S-POP/rGO example illustrates the mass-normalized logic: an approximately 0.04 mg cm^−2^ coating improved sulfur-conversion kinetics and retained activity at *E*/*S* = 5 µL mg^−1^.^59^ This route is preferable only when the catalytic and shuttle-control gains exceed the added impedance and inactive-mass penalty. Fig. 10 summarizes this prioritized architecture and its validation gates.

In the longer term, SACs may be combined with redox mediators, solid-state or quasi-solid electrolytes, protective lithium interfaces, and machine-learning-guided catalyst screening. Coordination-structure engineering in MOF-derived single atoms can guide this development, but the same design must be evaluated against synthesis reproducibility, catalyst cost, and earth-abundant metal availability.^100–102^ Mechanistic concepts from oxide catalysis, catalyst-size effects, and mixed-conductor solid-state Li–S interfaces are also relevant because they address sulfur conversion outside the narrow SAC literature.^103–105^ Material complexity should be justified by cell-level benefit. The most useful SAC will not be the one with the most elaborate coordination structure, but the one that improves energy density, cycle life, and manufacturability under practical constraints.

## 6. Conclusions

SACs anchored on nanostructured frameworks provide a rational path for improving Li–S batteries because they address several coupled limitations in sulfur chemistry. Isolated metal sites can bind LiPSs, lower sulfur-reduction barriers, promote Li_2_S nucleation, and assist Li_2_S oxidation. Nanostructured frameworks supply electron transport, ion access, sulfur accommodation, mechanical stability, and spatial control. The most effective systems combine these functions rather than relying on adsorption alone.

The literature shows clear progress. Co–N/G, V-on-N-doped graphene, Co-doped mesoporous carbon, asymmetric Co coordination, Zn and Ni single sites, Co-NPC separator coatings, and Pt-S-POP/rGO ultrathin separators demonstrate that metal identity, ligand field, framework porosity, and coating geometry can strongly influence cell behavior. Mechanistic studies have moved from simple LiPS trapping toward bidirectional conversion, Li_2_S decomposition, and product-surface autocatalysis. Quantitative examples show high capacities, low decay rates, and meaningful areal capacities when SACs are paired with conductive and porous frameworks.^106–112^

At the same time, the field remains limited by inconsistent testing and incomplete reporting. Many results are still obtained with low sulfur loading, excess electrolyte, excess lithium, and coin-cell formats. Some mechanistic descriptors are measured in simplified systems that do not capture product coverage, electrolyte depletion, and practical cell interfaces. Future studies should use standardized reporting, lean-electrolyte high-loading conditions, limited lithium, pouch-cell validation, and *operando* or post-cycling confirmation of single-site stability.

SACs are therefore best viewed as catalytic interface regulators rather than universal solutions. Their value depends on whether they enable reversible sulfur conversion under conditions that approach practical Li–S cell design. Progress will come from integrating atomic-scale coordination control with engineering-scale electrode architecture, electrolyte management, and transparent performance metrics.

## Author contributions

X. C. and T. L. contributed to the conceptualization and methodology of the review. X. C. conducted the literature investigation, curated and analyzed the comparative data, prepared the visualizations, and wrote the original draft. T. L. supervised the work, administered the project, acquired funding, and reviewed and edited the manuscript. All authors have read and agreed to the published version of the manuscript.

## Conflicts of interest

There are no conflicts to declare.

## Data Availability

No primary research results, software or code have been included and no new data were generated or analysed as part of this review.
